# Unraveling the differential impact of PAHs and dioxin-like compounds on AKR1C3 reveals the EGFR extracellular domain as a critical determinant of the AHR response

**DOI:** 10.1016/j.envint.2021.106989

**Published:** 2021-11-20

**Authors:** Christian Vogeley, Natalie C. Sondermann, Selina Woeste, Afaque A. Momin, Viola Gilardino, Frederick Hartung, Markus Heinen, Sophia K. Maaß, Melina Mescher, Marius Pollet, Katharina M. Rolfes, Christoph F.A. Vogel, Andrea Rossi, Dieter Lang, Stefan T. Arold, Motoki Nakamura, Thomas Haarmann-Stemmann

**Affiliations:** a IUF – Leibniz-Research Institute for Environmental Medicine, 40225 Düsseldorf, Germany; b King Abdullah University of Science and Technology (KAUST), Computational Bioscience Research Center (CBRC), Division of Biological and Environmental Sciences and Engineering (BESE), Thuwal 23955-6900, Saudi Arabia; c Department of Environmental Toxicology and Center for Health and the Environment, University of California, Davis, CA 95616, USA; d Bayer AG, Pharmaceuticals, Research Center, 42096 Wuppertal, Germany; e Centre de Biologie Structurale (CBS), INSERM, CNRS, Université de Montpellier, F-34090 Montpellier, France; f Department of Environmental and Geriatric Dermatology, Graduate School of Medical Sciences, Nagoya City University, Nagoya 467-8601, Japan

**Keywords:** Aldo-keto reductase 1C3, Aryl hydrocarbon receptor, Environmental pollutants, Epidermal growth factor receptor, Dioxin-like compounds, Polycyclic aromatic hydrocarbons

## Abstract

Polycyclic aromatic hydrocarbons (PAHs), dioxin-like compounds (DLCs) and structurally-related environmental pollutants may contribute to the pathogenesis of various diseases and disorders, primarily by activating the aryl hydrocarbon receptor (AHR) and modulating downstream cellular responses. Accordingly, AHR is considered an attractive molecular target for preventive and therapeutic measures. However, toxicological risk assessment of AHR-modulating compounds as well as drug development is complicated by the fact that different ligands elicit remarkably different AHR responses. By elucidating the differential effects of PAHs and DLCs on aldo–keto reductase 1C3 expression and associated prostaglandin D_2_ metabolism, we here provide evidence that the epidermal growth factor receptor (EGFR) substantially shapes AHR ligand-induced responses in human epithelial cells, i.e. primary and immortalized keratinocytes and breast cancer cells. Exposure to benzo[a]pyrene (B[a]P) and dioxin-like polychlorinated biphenyl (PCB) 126 resulted in a rapid c-Src-mediated phosphorylation of EGFR. Moreover, both AHR agonists stimulated protein kinase C activity and enhanced the ectodomain shedding of cell surface-bound EGFR ligands. However, only upon B[a]P treatment, this process resulted in an auto-/paracrine activation of EGFR and a subsequent induction of aldo–keto reductase 1C3 and 11-ketoreduction of prostaglandin D_2_. Receptor binding and internalization assays, docking analyses and mutational amino acid exchange confirmed that DLCs, but not B[a]P, bind to the EGFR extracellular domain, thereby blocking EGFR activation by growth factors. Finally, nanopore long-read RNA-seq revealed hundreds of genes, whose expression is regulated by B[a]P, but not by PCB126, and sensitive towards pharmacological EGFR inhibition. Our data provide novel mechanistic insights into the ligand response of AHR signaling and identify EGFR as an effector of environmental chemicals.

## 1. Introduction

The aryl hydrocarbon receptor (AHR) is a transcription factor that modulates gene expression in response to a variety of small molecular weight compounds, including numerous environmental pollutants of emerging concern, such as dioxins, dioxin-like polychlorinated biphenyls (PCBs) and polycyclic aromatic hydrocarbons (PAHs) (Murray et al., 2014; Rothhammer and Quintana, 2019; Vogel et al., 2020). Whereas AHR signaling driven by endogenous and dietary ligands, such as tryptophan metabolites and indoles, contributes to proper organ development and maintains cell and tissue homeostasis, a long-lasting activation of AHR by environmental chemicals may contribute to a variety of adverse health effects, including autoimmune and allergic inflammatory diseases, endocrine disruption and cancer (Murray et al., 2014; Roman et al., 2018; Rothhammer and Quintana, 2019; Stockinger et al., 2021; Vogel et al., 2020). Accordingly, AHR is considered an attractive target for the development of novel preventive and therapeutic measures. However, drug development as well as the toxicological risk assessment of AHR-modulating compounds is considerably complicated by a remarkable ligand-specificity of AHR signaling and associated outcome. An illustrating example is the opposing impact of dioxin-like compounds (DLCs) and PAHs on T cell differentiation and the development and worsening of allergic inflammatory diseases, such as asthma and atopic dermatitis. Whereas a chronic exposure to 2,3,7,8-tetrachlorodibenzo-*p*-dioxin (TCDD) and related DLCs induces immunosuppressive regulatory T cells (Funatake et al., 2005; Quintana et al., 2008), exposure to PAHs and PAH-rich airborne particulate matter suppresses regulatory T cell differentiation (Nadeau et al., 2010; O’Driscoll et al., 2018) and promotes the expansion of Th2 and Th17 cells (Castañeda et al., 2018; Hidaka et al., 2017; Hong et al., 2016; O’Driscoll et al., 2018; Weng et al., 2018; Wong et al., 2018; Xia et al., 2015). Accordingly, an exposure to free and particle-bound PAHs is associated with an increased risk for asthma and atopic dermatitis (Dijkhoff et al., 2020; Lag et al., 2020), whereas elevated systemic levels of DLCs correlate with a decreased incidence of atopic diseases (Nakamoto et al., 2013; Ye et al., 2018). Furthermore, transcriptome analyses revealed that different AHR ligands induce a different pattern of gene expression even within the same tissue or cell-type (Kopec et al., 2010; Nault et al., 2013; Souza et al., 2016). The molecular mechanisms responsible for this ligand specificity of AHR signaling are not well understood.

Inactive AHR is part of a cytosolic multiprotein complex consisting of a heat-shock protein 90 dimer, AHR-interacting protein, the co-chaperone p23 and cytoplasmic tyrosine kinase c-Src. Upon ligand-binding, this complex dissociates and AHR translocates into the nucleus, dimerizes with AHR nuclear translocator (ARNT) and binds to xenobiotic-responsive elements (XRE) in the enhancer region of target genes, e.g. encoding cytochrome P450 (CYP) 1A1 and CYP1B1, to induce their transcription (Murray et al., 2014; Roman et al., 2018; Rothhammer and Quintana, 2019; Stockinger et al., 2021; Vogel et al., 2020). In addition, there are multiple non-canonical functions of AHR, for instance involving a modulation of epidermal growth factor receptor (EGFR) and NF-E2-related factor 2 (NRF2) signaling pathways, that may shape the outcome of AHR signaling (Roman et al., 2018; Vogel et al., 2020).

The expression of human aldo–keto reductase (AKR) 1C3 is differentially regulated by prototypical AHR ligands, i.e. upregulated in response to PAHs but unaffected by TCDD treatment (Burczynski et al., 1999). AKR1C3 is a cytosolic NADPH-dependent oxidoreductase that catalyzes the biosynthesis of 17β-estradiol and testosterone and the reduction of prostaglandin (PG) D_2_ and PGH_2_ to 9α,11β-PGF_2_ and PGF_2_α, respectively (Liu et al., 2020; Penning, 2019). Accordingly, AKR1C3 contributes to the pathogenesis of various types of cancer, inflammatory diseases and endocrine disorders (Liu et al., 2020; Penning, 2019). Despite its clinical relevance, the regulation of AKR1C3, particularly in response to environmental factors, is not well understood.

Here, we elucidated the molecular mechanisms underlying the ligand-specific differences in the AHR response of human epithelial cells. We identified a complex AHR- and EGFR-dependent signaling pathway which is initiated by PAH treatment to modulate the expression of hundreds of genes. Although initially stimulating similar signaling events, dioxin-like compounds (DLCs) interrupted this pathway by binding to the extracellular domain (ECD) of EGFR and inhibiting its activation by growth factors.

## 2. Material and methods

### 2.1. Cell culture

Normal human epidermal keratinocytes (NHEKs) obtained from PromoCell (Heidelberg, Germany) were cultured in Keratinocyte Growth Medium 2 (PromoCell). HaCaT, HaCaT-AHR-KO, HaCaT-CYP1A1-KO and HaCaT-NRF2-KO keratinocytes were cultured in DMEM low glucose (1 g/l) medium (PAN Biotech, Aidenbach, Germany) and supplemented with 10% FBS and antibiotics/antimycotics (PAN Biotech). Stable AHR-knockdown HaCaT keratinocytes (HaCaT-shAHR) and respective empty vector control cells (HaCaT-EV) were cultured in regular HaCaT medium supplemented with 0.68 mg/ml G418 (Carl Roth, Karlsruhe, Germany). MCF-7 and MCF-7-AHR-KO cells were cultured in DMEM high glucose (4.5 g/l) medium (PAN Biotech) supplemented with 10% FBS and antibiotics/antimycotics. The generation and characterization of HaCaT-shAHR and HaCaT-EV cells (Fritsche et al., 2007) and MCF-7-AHR-KO cells (Vogel et al., 2021) has been previously described. HepG2 cells were cultured in RPMI 1640 containing 10% FBS and antibiotics/antimycotics (PAN Biotech). All cells were kept in a humidified atmosphere of 5% CO_2_ at 37 °C.

### 2.2. Chemicals and treatment

Benzo[a]pyrene, benzo[k]fluoranthene, bosutinib, 1,2-dithiole-3-thione, flufenamic acid, MG132, PP2, Ro-31–8220 and hydrogen peroxide were purchased from Sigma-Aldrich (Taufkirchen, Germany), BPIQII, SR11302 and T-5224 from Cayman Chemicals (Ann Arbor, MI), and CH223191, cobimetinib and glutathione from Selleckchem (Houston, TX). Marimastat was purchased from Santa Cruz Biotechnology (Dallas, TX) and PD153035 from Absource Diagnostics (Munich, Germany). 3,3′,4,4′,5-Pentachlorobiphenyl (PCB126) and 2,3′,4,4′,5-pentachlorobiphenyl (PCB118) were bought from LGC Standards (Wesel, Germany) and 2,3,7,8-tetrachlorodibenzo-*p*-dioxin from Amchro (Hattersheim am Main, Germany). Prostaglandin D_2_, AREG, TGFα and EGF were purchased from PeproTech (Rocky Hill, NY). Hydrogen peroxide, glutathione and the three EGFR ligands were dissolved or diluted in water, the other compounds in DMSO. Treatment time and applied concentrations of the chemicals and human recombinant proteins is indicated in the figure legends.

### 2.3. Quantitative real-time PCR

Total RNA was isolated and transcribed into cDNA by using the GenUP Total RNA Kit (Biotechrabbit, Hennigsdorf, Germany) and a M−MLV reverse transcriptase (Promega, Walldorf, Germany). Quantitative real-time PCR analyses were carried using the QuantiFast SYBR Green PCR Kit and a Corbett-Rotor Gene 300 light cycler (Qiagen, Hilden, Germany). Oligonucleotide sequences are listed in supplementary table 1a.

### 2.4. Transient transfection of siRNA and plasmid DNA

Transient transfection of HaCaT cells with ARNT-targeted and non-silencing siRNA (Santa Cruz Biotechnology, Dallas, TX) was done using the INTERFERin reagent (Polyplus Transfection, Illkirch, France). JetPEI DNA Transfection Reagent (Polyplus) was used for transient transfection with plasmid DNA.

### 2.5. SDS-PAGE/Western blot analysis

Cells were lysed in RIPA buffer on ice and subsequently centrifuged for 5 min at 4 °C at maximum speed. Protein samples were separated by 10% and 12% SDS-polyacrylamide gel electrophoresis and blotted onto PVDF membranes (GE Healthcare, Freiburg, Germany). Blots were blocked with 5% skim milk or bovine serum albumin in TBS-Tween-20 (0.1%) for 1 h at room temperature and subsequently incubated overnight at 4 °C with primary antibodies. Blots were washed and then incubated for 1 h with a 1:5000 dilution of HRP-conjugated secondary antibodies (Cell Signaling Technology, Leiden, The Netherlands) in 5% bovine serum albumin in TBS-Tween-20 (0.1%) at room temperature. Bands were visualized using WesternBright ECL HRP substrate (Advansta, San Jose, CA) and a C-DiGit Western Blot Scanner (LI-COR Biotechnology, Lincoln, NE). Primary antibodies are listed in supplementary table 1b.

### 2.6. Incubation of AKR isoforms with PGD_2_

The incubation mixtures contained 50 mM phosphate buffer (pH 7.4) with 1 mM EDTA, 400 pmol/mL of recombinant AKRs and a NADPH generating system consisting of 1 mM NADP+, 5 mM glucose 6-phosphate, 0.5 IU of glucose 6-phosphate dehydrogenase and 2 μM PGD_2_. After a preincubation of 2 min at 37 °C, the reaction was initiated by the addition of the NADPH generating system and was allowed to continue at 37 °C for up to 60 min. Incubations were terminated with the addition of 30% (v/v) acetonitrile. Protein was precipitated by centrifugation, and the supernatants were subjected to LC-MS analysis.

### 2.7. LC–MS analyses

Incubations were analyzed using reversed-phase HPLC with a Synergi Hydro-RP 4 μm, 150 × 2 mm column (Phenomenex, Aschaffenburg, Germany) and gradient elution using 10 mM ammonium formate and acetonitrile containing 0.1% formic acid as solvents. The HPLC was coupled to a high-resolution Orbitrap Fusion™ Tribride™ mass spectrometer (Thermo Fisher Scientific, Bremen, Germany) operated in full-scan mode. Selectivity of the analytes was gained by extracting a very narrow mass range in the order of 10–20 ppm of the exact mass of the analyte. 9α,11β-PGF_2_ was identified using authentic standard.

### 2.8. Generation of CRISPR/Cas9 and CRISPR/Cas12 mutated cells

The generation of CYP1A1-KO and NRF2-KO HaCaT keratinocytes was carried out as described previously (Vogel et al., 2021). The respective gRNAs (Fig. S1) were designed using the CRISPR design tool CHOPCHOP (http://chopchop.cbu.uib.no/) and cloned into a modified version of the PX458 plasmid available at Addgene (#48138). The resulting bicistronic vectors each encoded the respective gRNA and the Cas9 nuclease. AHR-KO HaCaT keratinocytes were generated delivering a ribonucleoprotein consisting of a Cas12 protein (IDT) in complex with a targeting gRNA. Activity of the gRNAs and their efficiency were assessed *via* high resolution melt analysis. HaCaT cells were transfected with nuclease plasmids in antibiotic-free medium in a 12-well plate using FuGENE HD (Roche, Mannheim, Germany) or the NEON electroporation system (Thermo Fisher Scientific). After 48 h, cells were sorted (FACS or MACS) and plated as single cells in a 96-well plate and duplicated after a week. Clones were lysed in proteinase K and genotyped using high-resolution melt analysis, SANGER sequencing or deep sequencing using a MiSeq Illumina (San Diego, CA) (Ramachandran et al., 2021).

### 2.9. PKC activity assay

PKC activity was analyzed by using the PKC Kinase Activity Kit (Enzo Life Sciences, Loerrach, Germany). Treatment medium was removed from the cells were washed with PBS twice. Lysates were prepared using 100 μl MOPS lysis buffer and samples were analyzed according to manufacturer’s instructions.

### 2.10. ELISA-based quantification of growth factors

The concentration of TGFα and AREG in cell culture supernatant was determined by using respective Quantikine ELISA Kits (Bio-Techne, Wiesbaden, Germany). Therefore, the supernatant from treated cells was transferred into microtubes and centrifuged at 5000 rcf at 4 °C for 5 min. The supernatant was analyzed according to manufacturer’s instructions.

### 2.11. EGFR docking analyses

Crystal structures of the extracellular domain of EGFR in an inactive complex with EGF (PDB ID:1nql) and dimeric (2:2) complex of EGFR and EGF (PDB ID: 1ivo) were used to perform docking analyses. Ligand structures for TCDD (PubChem CID: 15625), PCB126 (PubChem CID: 63090), 1,2,3,7,8-pentachlorodibenzo-*p*-dioxin (PCDD; PubChem CID: 38439), 1,2,3,4,7,8-hexachlorodibenzo-*p*-dioxin (HCDD; PubChem CID: 38251), 2,3,7,8-tetrachlorodibenzofuran (TCDF; PubChem CID: 39929), 3,3^′^,4,4^′^-tetrachlorobiphenyl (PCB77; PubChem CID: 36187), PCB118 (PubChem CID: 35823), and B[a]P (PubChem CID:125144) were downloaded from PubChem database (Kim et al., 2016) in SD format. The EGF domain was removed from the protein structure using PyMol (www.pymol.org), and ligand structures were converted to PDB format using OpenBabel 2.3.1 (O’Boyle et al., 2011). Flexible docking was performed using AutoDock 4.2 (Goodsell et al., 1996) as previously described (Hawerkamp et al., 2019), except that the size for the grid box (x,y,z points) were set to 51 × 39 × 58, while centers for the grid were designated at X = 113.38, Y = 65.95, Z = 39.94 dimensions. The final docking poses were analyzed with PyMol (www.pymol.org).

### 2.12. EGFR internalization assay

Internalization of EGFR was investigated using a High-Content-Screening method adapted from Wang *et al.* (Wang and Xie, 2007). Cells were treated with 200 ng/ml EGF-AF555 (Thermo Fisher Scientific, Waltham, MA) for 2.5 min on ice. Afterwards the test compounds were added and incubated for another 2.5 min on ice. Internalization was enabled by incubating the cells at 37 °C and 5 % CO_2_ for 25 min. The cells were fixed with 4 % paraformaldehyde. The membrane was stained with Wheat Germ Agglutinin, Oregon Green™ 488 Conjugate and the nuclei with Hoechst33342 (both Thermo Fisher Scientific). HCS analysis was performed using a Cellomics ArrayScan VTI (Thermo Fisher Scientific) and the images were analyzed with the HCS Studio Cellomics Scan software (version 6.6.0).

### 2.13. EGF-EGFR AlphaLISA binding assay

A potential disturbance of the binding of EGF to EGFR by the test compounds was analyzed by using the cell-free EGF/EGFR AlphaLISA Binding Kit (PerkinElmer, Waltham, MA).

### 2.14. Site-directed mutagenesis

For the generation of point-mutated EGFR variants, the pCMV3-EGFR^wt^ plasmid (Sino Biological, Eschborn, Germany) was used as template. Briefly, a high fidelity Q5 polymerase (New England Biolabs, Ipswich, MA) was used to amplify the whole plasmid with complementary primer pairs, carrying the desired mutation in the form of mismatches to the original plasmid. PCR conditions were: 94 °C for 2 min, 21 cycles of 94 °C (30 sec), 55 °C (1 min) and 68 °C (30 sec/kb). After three cycles, the oligonucleotides for the introduction of the respective point-mutations were added; primer sequences are listed in supplementary table 1c. Afterwards, the PCR mix was treated with DpnI endonuclease (New England Biolabs) to remove parental DNA. The successful introduction of point-mutations was validated by Sanger Sequencing.

### 2.15. Measurement of DNA synthesis

DNA synthesis was assessed using a colorimetric BrdU incorporation assay (Sigma-Aldrich, Taufkirchen, Germany). Briefly, HaCaT keratinocytes were seeded on 96-well-plates in quintuplicate (2×10^4^ per well) and starved overnight. Approximately 12 h later, the cells were treated with the respective test substances and BrdU labeling solution for 4 h. Subsequently, the assay was carried out according to the manufacturers protocol. Finally, after 15 min incubation with substrate solution, absorbance was measured at 370 nm (reference wavelength 492 nm) using the Infinite 200 PRO plate-reader (Tecan, Maennedorf, Switzerland).

### 2.16. ROS measurement

Cells were incubated with 100 μM DCF-diacetate diluted in PBS *post* treatment for 30 min at 37 °C and 5 % CO_2_. Afterwards, the DCF-diacetate dilution was removed and the fluorescence at 485_ex_/535_em_ nm was measured using the Infinite 200 PRO plate-reader (Tecan).

### 2.17. CYP1A1 activity assay

Measurement of the deethylation of 7-ethoxyresorufin in living monolayer cultures was carried out as described previously (Frauenstein et al., 2015).

### 2.18. Library preparation and sample loading for long-read nanopore RNA-sequencing

Quality of isolated RNA was assessed using the High Sensitivity RNA Screen Tape System (Agilent Technologies, Santa Clara, CA). Reverse transcriptase and multiplexing of the samples were performed with the PCR cDNA Barcoding Kit (SQK-PCB109, Oxford Nanopore Technologies, Oxford, United Kingdom) using 50 ng total RNA. Quantity of amplified cDNA was determined with the Qubit™ 4 Fluorometer (Invitrogen, Carlsbad, CA) and the range of fragment size was examined using the Agilent D1000 SreenTape assay (Agilent Technologies). The Flowcell (FLO-MIN106) was prepared with the FlowCell Priming Kit (EXP FLP002, Oxford Nanopore Technologies) and equal amounts of barcoded cDNA was loaded. Sequencing was carried out with a MinION (MN33710) using the MinKNOW software (v.21.02.1) over a period of 72 h.

### 2.19. RNA-Seq data analysis

Raw fast5 reads were base-called and demultiplexed using Guppy (v4.5.4 + 66c1a7753). Reads were aligned to the reference genome (GRCh38.94) using Minimap2 (Li, 2018). Uniquely mapped reads were summarized with the featureCounts function of the R (v4.0.3) package Rsubread (v1.32.2) (Liao et al., 2019). Differential expression analysis was performed with DESeq2 (v1.22.1) (Love et al., 2014) and cluster-Profiler (v4.0.0) (Yu et al., 2012) was used for gene set enrichment analysis.

### 2.20. Multiple sequence alignment

The multiple alignment of the N-terminal 420 amino acids of the EGFR protein (mature) from various mammalian species was carried out using Clustal Omega (v1.2.4) (Sievers et al., 2011) and the following NCBI Reference Sequences: *Homo sapiens*, NP_005219.2; *Macaca mulatta*, XP_014988922.2; *Callithrix jacchus*, XP_035109337.1; *Mus musculus*, NP_997538.1; *Rattus norvegicus*, AAF14008.1; *Oryctolagus cuniculus*, XP_008260065.1; *Sus scrofa*, NP_999172.1; *Ovis aries*, XP_014957685.2; *Felis catus*, XP_006929148.1; *Canis lupus dingo*, XP_025305356.1.

### 2.21. Statistical analysis

All data shown are mean (±standard deviation) from three or more independent experiments, if not indicated otherwise. Differences were considered significant at p ≤ 0.05. A comparison of two groups was performed by unpaired, two-tailed Student’s *t* test. A comparison of multiple groups was conducted with analysis of variance followed by a Tukey *post hoc* analysis to correct for multiple comparison.

## 3. Results

### 3.1. B[a]p but not PCB126 induces AKR1C3 expression in an AHR-dependent manner

**Fig. 1.**
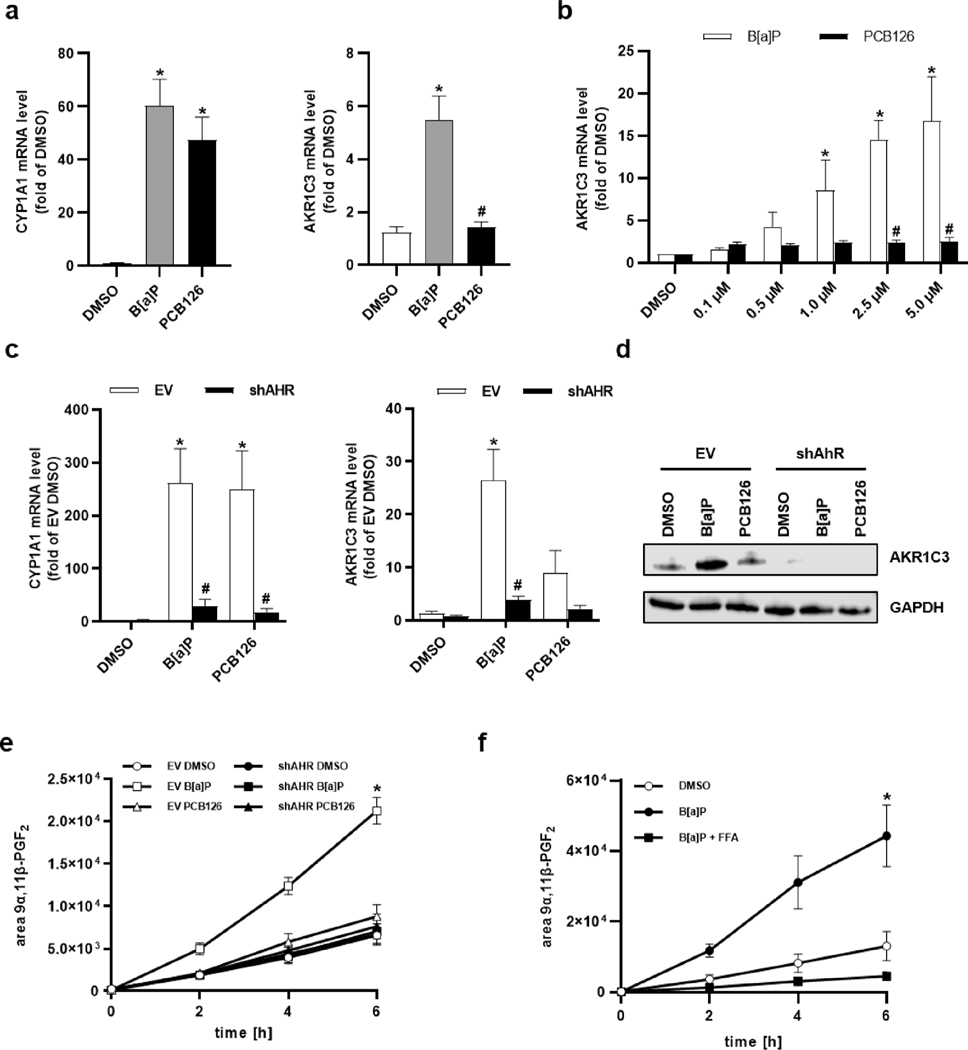
B[a]P induces AKR1C3 expression in an AHR-dependent manner. **a** qRT-PCR analyses of *CYP1A1* and *AKR1C3* in NHEKs stimulated with B[a]P (2.5 μM), PCB126 (1 μM) or solvent (0.1 % DMSO) for 24 h. n = 9. *, p ≤ 0.05 compared to DMSO; #, p ≤ 0.05 compared to B[a]P. **b** qRT-PCR analysis of *AKR1C3* in HaCaT keratinocytes stimulated as indicated for 24 h. n = 6. *, p ≤ 0.05 compared to DMSO control, #, p ≤ 0.05, compared to B[a]P of the same concentration. **c** qRT-PCR analyses of *CYP1A1* and *AKR1C3* in HaCaT-shAHR and HaCaT-EV keratinocytes exposed to 2.5 μM B[a]P, 1 μM PCB126 or 0.1 % DMSO for 24 h. n = 4. *, p ≤ 0.05 compared to EV DMSO, #, p ≤0.05 compared to EV B[a]P. **d** Western blot analyses of AKR1C3 protein content in HaCaT-shAHR and HaCaT-EV keratinocytes stimulated as described in **c**. GAPDH level served as loading control. n = 3, representative picture. **e** LC-MS analyses of supernatants derived from HaCaT-shAHR and HaCaT-EV cells stimulated with 0.1 % DMSO, 1 μM PCB126 or 2.5 μM B[a]P for 24 h. Afterwards, cells were treated with 1 μM PGD_2_ in conditioned medium and supernatants were collected at indicated time points. n = 3. *, p ≤ 0.05 compared to DMSO. **f** LC-MS analyses of the supernatants of HaCaT cells treated with 0.1 % DMSO and 2.5 μM B[a]P for 24 h. In addition, HaCaT cells pretreated for 23 h with 2.5 μM B[a]P were co-exposed for 1 h to 50 μM flufenamic acid (FFA) and B[a]P. Subsequently, cells were treated with 1 μM PGD2 in conditioned medium and the supernatant was collected at indicated time points. Supernatants were collected at indicated time points. n = 3. *, p ≤ 0.05 compared to DMSO.

Treatment of NHEKs with 2.5 μM of the PAH benzo[*a*]pyrene (B[a]P) and 1 μM of the DLC 3,3^′^,4,4^′^,5-pentachlorobiphenyl (PCB126) for 24 h increased *CYP1A1* copy numbers to a similar extent (Fig. 1a). In contrast, the *AKR1C3* transcript level was only induced by B[a]P but not by PCB126 treatment. Dose- and time course studies in immortalized human HaCaT keratinocytes confirmed a strict AHR ligand-dependent regulation of *AKR1C3* (Fig. 1b, S2a). Accordingly, exposure to benzo [*k*]fluoranthene (B[k]F) induced the expression of both *CYP1A1* and *AKR1C3*, whereas TCDD treatment only increased the copy numbers of *CYP1A1* (Fig. S2b). Gene and protein expression analyses of B[a]P- and PCB126-treated *AHR*-knockdown (HaCaT-shAHR) and CRISPR/Cas12-engineered *AHR*-knockout (HaCaT-AHR-KO) keratinocytes, *AHR*-knockout human MCF-7 breast cancer cells (MCF-7-AHR-KO) and respective AHR-proficient controls confirmed a ligand-specific and AHR-dependent upregulation of AKR1C3 (Fig. 1c,d, S2c,d). Further qPCR analyses revealed a similar expression pattern for other AKR1 isoforms, i.e. *AKR1C2* and *AKR1B10* (Fig. S2e). Hence, in contrast to PCB126, a treatment of human epithelial cells with B[a]P induces the expression of *AKR1C3* and related isoforms in an AHR-dependent manner.

### 3.2. B[a]p but not PCB126 stimulates PGD_2_ metabolism

Next, we assessed potential alterations in AKR1C3 enzyme activity and analyzed the 11-ketoreduction of PGD_2_. An incubation of 2 μM PGD_2_ with microsomal preparations of heterologously expressed human AKR1C isoforms revealed an efficient and NADPH-dependent reduction of PGD_2_ to 9α,11β-PGF_2_ by AKR1C3 (Fig. S2f). AKR1C1 and AKR1C2 were roughly 10-times less effective in reducing PGD_2_, whereas the liver-specific isoform AKR1C4 only had a minor impact. A pretreatment of AHR-proficient cells with B[a]P resulted in an enhanced formation of 9α,11β-PGF_2_ as compared to solvent controls (Fig. 1e). This effect was neither observed in cells pretreated with PCB126 nor in B[a]P-exposed HaCaT-shAHR keratinocytes. AHR-dependency of the B[a]P-induced generation of 9α,11β-PGF_2_ was confirmed in NHEKs cotreated with the AHR antagonist CH223191 (Fig. S2g). Moreover, a co-treatment of HaCaT cells and NHEKs with B[a]P and the AKR1C3 inhibitor flufenamic acid (FFA) blunted the B[a]P-stimulated reduction of PGD_2_ to 9α,11β-PGF_2_ (Fig. 1f, S2g). Taken together, these data provide evidence that in contrast to PCB126 an exposure of human primary and immortalized keratinocytes to B[a]P stimulates the AKR1C3-catalyzed metabolism of PGD_2_ in an AHR-dependent manner.

### 3.3. B[a]p induces AKR1C3 expression independently from canonical AHR signaling

**Fig. 2.**
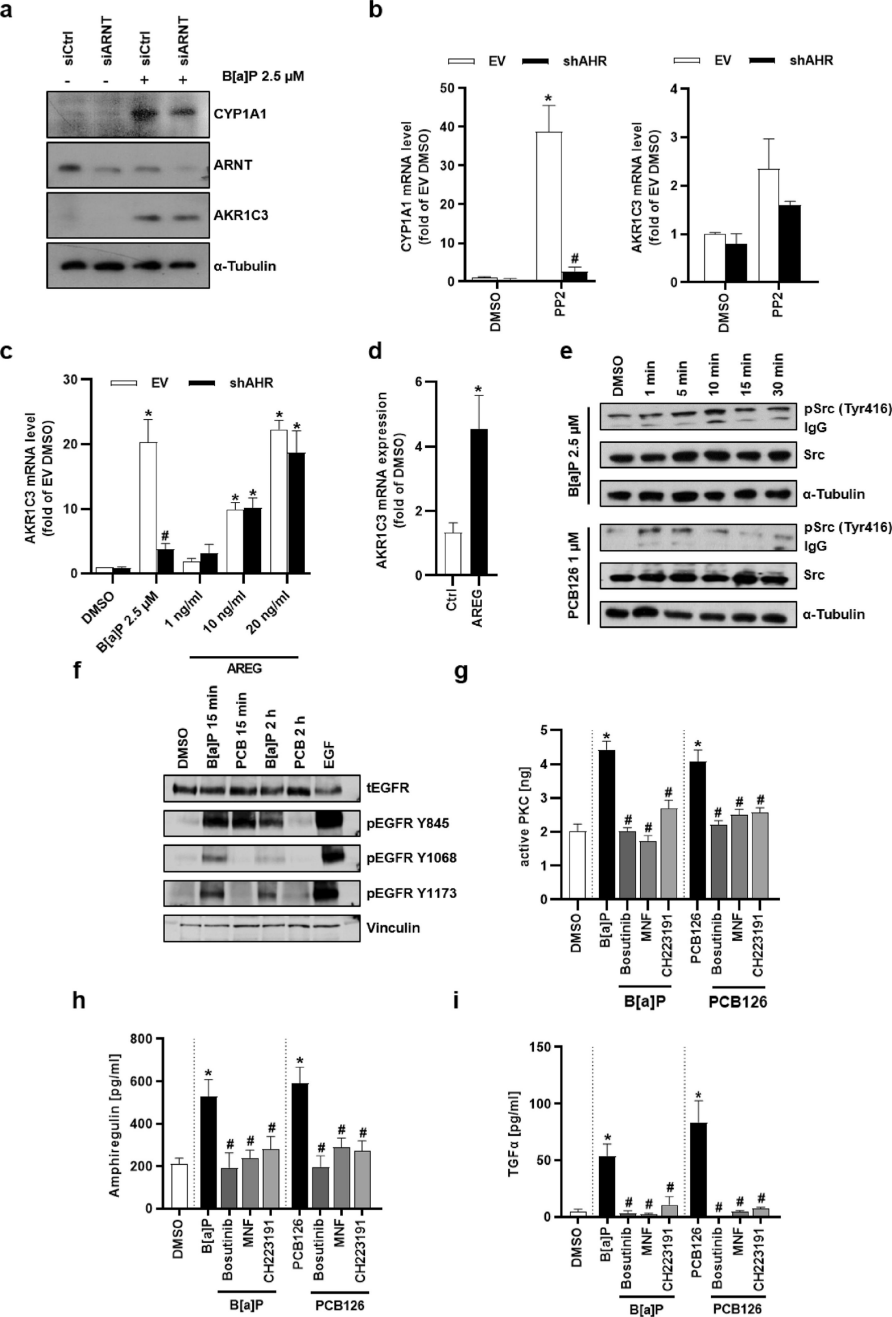
B[a]P stimulates AKR1C3 expression in an EGFR-dependent manner. **a** HaCaT keratinocytes were transiently transfected with ARNT-targeted or non-silencing siRNA for 24 h. Next, cells were treated with 2.5 μM B[a]P or solvent for another 24 h. The protein level of CYP1A1, ARNT and AKR1C3 was detected by western blot analyses, α-tubulin was used as loading control. n = 2, representative pictures. **b** qRT-PCR analyses of *CYP1A1* and *AKR1C3* in HaCaT-shAHR and HaCaT-EV cells treated with 10 μM PP2 or 0.1 % DMSO for 24 h. n = 4. *, p ≤ 0.05 compared to EV DMSO, #, p ≤ 0.05 compared to EV B[a]P. **c** qRT-PCR analyses of *CYP1A1* and *AKR1C3* in HaCaT-shAHR and HaCaT-EV keratinocytes treated as indicated for 24 h. n = 4–7. *, p ≤ 0.05 compared to EV DMSO, #, p ≤ 0.05 compared to EV B[a]P. **d** NHEKs were treated with 20 ng/ml AREG for 24 h and *AKR1C3* transcript level were analyzed by qRT-PCR. **e** Western blot analyses of SRC and its phosphorylated form (Y416). HaCaT keratinocytes were treated with B[a]P (2.5 μM), PCB126 (1 μM) or 0.1 % DMSO for the indicated time. n = 3, representative pictures. **f** Phosphorylation of EGFR in HaCaT keratinocytes treated with B[a]P (2.5 μM) or PCB126 (1 μM) was examined by western blot analysis. Cells were treated for 15 min and 2 h. DMSO (0.1 %) served as solvent control, EGF (10 ng/ml) as positive control. n = 3, representative picture. **g** Levels of activated PKC were quantified using a non-radioactive protein kinase activity assay. HaCaT keratinocytes were pre-treated with Bosutinib (1 μM), MNF (20 μM), CH223191 (10 μM) or 0.1 % DMSO for 1 h. Afterwards, cells were stimulated with B[a]P (2.5 μM) or PCB126 (1 μM) and 0.1 % DMSO. After 2 h, cells were lysed and PKC activity was determined. ELISA-based quantification of **h** AREG and **i** TGFα in the cell culture supernatants. Cells were treated as described in **g.** Supernatants were collected 2 h after treatment. n = 4. *, p ≤ 0.05 compared to DMSO, #, p ≤ 0.05 compared to either B[a]P or PCB126 treated cells respectively.

A transient RNAi-mediated knockdown of ARNT dampened the B[a] P-induced upregulation of CYP1A1 by approximately 40% but did not affect the B[a]P-mediated induction of AKR1C3 protein (Fig. 2a), indicating that AKR1C3 is not part of the AHR/XRE gene battery. Treatment of HaCaT cells with PP2, a pharmacological inhibitor of Src family kinases and potent AHR agonist (Frauenstein et al., 2015), elevated the transcript numbers of *CYP1A1* but not of *AKR1C3* (Fig. 2b), suggesting an upregulation of *AKR1C3* through the non-canonical c-Src- and EGFR-dependent signaling pathway (Dong et al., 2011; Fritsche et al., 2007). This was supported by the observation that treatment with the EGFR ligand amphiregulin (AREG) increased *AKR1C3* copy numbers independently of AHR (Fig. 2c). An AREG-inducible expression of *AKR1C3* was also confirmed in NHEKs (Fig. 2d).

### 3.4. Treatment with B[a]P but not PCB126 results in a biphasic activation of EGFR

**Fig. 3.**
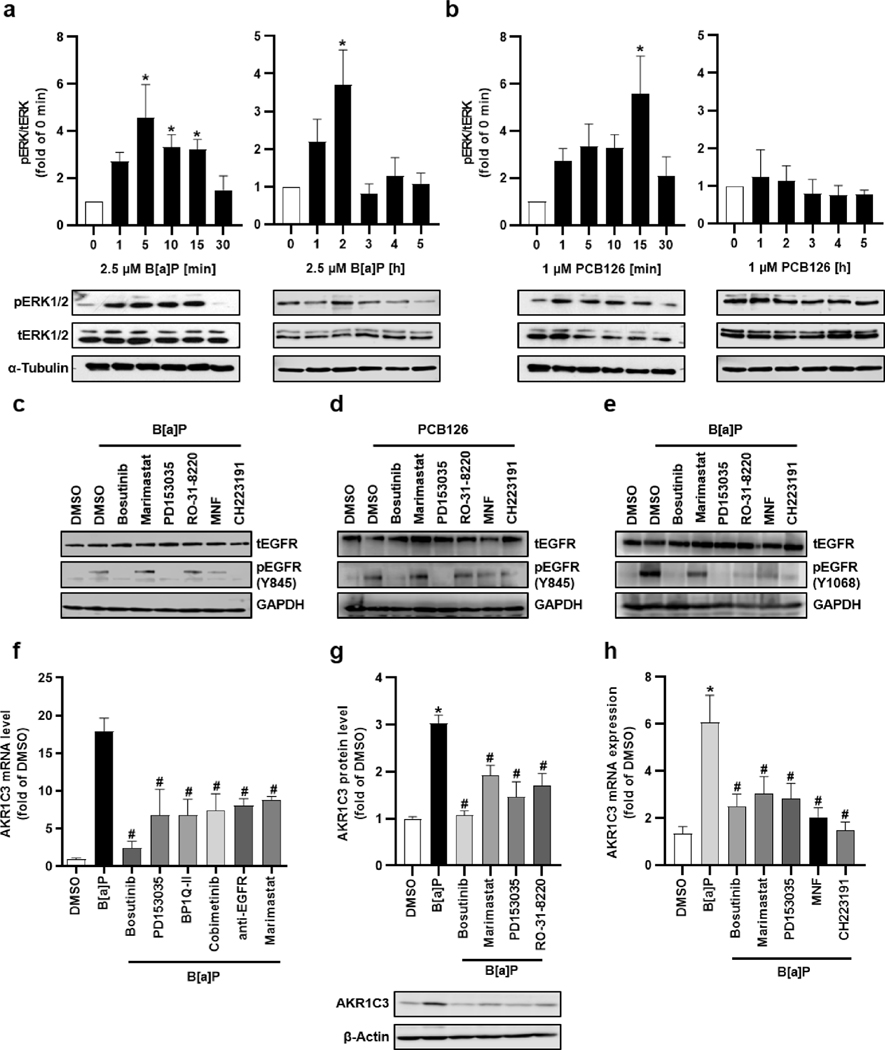
B[a]P stimulates AKR1C3 expression through a non-canonical signaling pathway. Western blot analyses of ERK1/2 phosphorylation upon exposure to **a** B[a]P (2.5 μM) and **b** PCB126 (1 μM) for the indicated time points. In the upper panel the densitometric quantification, in the lower panel representative blots are shown. n = 3 – 5. *, p ≤ 0.05 compared to DMSO. Western blot analysis of HaCaT cells treated with **c** B[a]P (2.5 μM) and **d** PCB126 (1 μM) for 15 min or **e** B[a]P (2.5 μM) for 2 h. In parallel, cells were co-treated with bosutinib (1 μM), marimastat (1 μM), PD153035 (1 μM), RO-31–8220 (1 μM), MNF (20 μM), CH223191 (10 μM) or DMSO (0.1 %). In **c** and **d** EGFR phosphorylation at residue Y845 and in **e** EGFR phosphorylation at residue Y1068 was examined. All results were normalized to total EGFR, GAPDH was used as loading control. n = 3, representative pictures. **f** qRT-PCR analyses of *AKR1C3* in HaCaT keratinocytes. The cells were treated with B[a]P (2.5 μM) in the absence and presence of either Bosutinib (1 μM), PD153035 (1 μM), BP1-QII (1 μM), Cobimetinib (1 μM), EGFR-blocking antibody (4 μg/ml), Marimastat (1 μM) or DMSO (0.1 %) for 24 h. n = 3 – 6. *, p ≤ 0.05 compared to DMSO, #, p ≤ 0.05 compared to B[a]P. **g** Western blot analysis of AKR1C3 protein level in HaCaT keratinocytes. Cells were treated as indicated at the concentrations depicted in **f** for 24 h. In the lower panel a representative blot, in the upper panel the densitometric quantification is shown. n = 3. *, p ≤ 0.05 compared to DMSO, #, p ≤ 0.05 compared to B[a]P. **h** qRT-PCR analyses of *AKR1C3* in NHEKs treated for 24 h with B[a]P (2.5 μM) in the absence and presence of either Bosutinib (1 μM), PD153035 (1 μM), Marimastat (1 μM), MNF (20 μM), CH223191 (10 μM) or DMSO (0.1 %). Additional cells were treated with 20 ng/ml AREG. n = 7. *, p ≤ 0.05 compared to DMSO, #, p ≤ 0.05 compared to B[a]P.

In line with earlier reports (Bessede et al., 2014; Dong et al., 2011), AHR activation by B[a]P and PCB126 resulted in a rapid phosphorylation of c-Src Y416 (Fig. 2e). This was accompanied by a phosphorylation of EGFR residue Y845, a substrate of c-Src (Chen et al., 2016), after 15 min of treatment with both AHR agonists (Fig. 2f). Upon B[a]P treatment, an additional phosphorylation of EGFR at residues Y1068 and Y1173 was observed, indicating an EGFR ligand-mediated activation and subsequent autophosphorylation of the receptor tyrosine kinase (RTK) (Chen et al., 2016). After elongating the treatment time (2 h), these two residues were still phosphorylated in B[a]P-treated but not in PCB126-exposed cells (Fig. 2f). Importantly, c-Src may also activate PKC (Joseloff et al., 2002), which subsequently stimulates metalloproteases, such as A Disintegrin And Metalloprotease (ADAM) 17, to shed the ectodomain of cell-surface bound EGFR ligands, including AREG and transforming growth factor α (TGFα) (Chen et al., 2016; Seals and Courtneidge, 2003). Indeed, a 2 h exposure of HaCaT cells to B[a]P and PCB126 stimulated both PKC enzyme activity (Fig. 2g) and the release of AREG and TGFα in an AHR- and c-Src-dependent manner (Fig. 2h,i). Hence, although both AHR ligands stimulate the phosphorylation of c-Src and EGFR Y845 and activate PKC and associated shedding of EGFR ligands, only B[a]P mimicked an EGF-induced EGFR autophosphorylation (Y1068, Y1173). Accordingly, we noted AHR ligand-specific differences in the phosphorylation of EGFR downstream extracellular-regulated kinase 1/2 (ERK). B[a]P exposure of HaCaT cells caused a biphasic phosphorylation of ERK, with peaks observed after 5 and 120 min (Fig. 3a). On the contrary, PCB126 treatment only stimulated ERK phosphorylation at an early timepoint, i.e. after 15 min (Fig. 3b). Further experiments revealed that the early phosphorylation of EGFR residue Y845 induced by both B[a]P and PCB126 was sensitive towards AHR antagonists (MNF, CH223191), the Src kinase inhibitor bosutinib and the EGFR blocker PD153035, but not towards inhibition of PKC (Ro-31–8220) and metalloproteases (marimastat) (Fig. 3c,d). In contrast, the B[a]P-induced phosphorylation of EGFR residue Y1068 after 2 h of treatment was attenuated by all inhibitors tested (Fig. 3e). An analysis of B[k]F-treated HaCaT cells confirmed an enhanced phosphorylation of EGFR (Y1068) after 2 h, which was sensitive towards pharmacological inhibition of Src kinase and PKC (Fig. S3).

### 3.5. Inhibition of non-canonical AHR signaling abrogates the B[a]P-mediated AKR1C3 induction

So far, our data provide evidence that B[a]P and PCB126 have a differential impact on EGFR activation, with B[a]P causing a longer lasting activation through an extracellular stimulus. This was supported by co-treatment of HaCaT cells, NHEKs and MCF-7 cells with various pharmacological inhibitors, revealing that the B[a]P-induced upregulation of *AKR1C3* involved c-Src (bosutinib), PKC (RO-31–8220), metalloprotease activity (marimastat), EGFR (PD153035, BPIQII) and MEK (cobimetinib) (Fig. 3f–h, Fig. S4). Importantly, the B[a]P-triggered induction of *AKR1C3* was also reduced by the addition of an EGFR-blocking antibody in HaCaT cells (Fig. 3f) as well as by co-exposure to PCB126 in MCF-7 cells (Fig. S4). The latter observation indicates that although stimulating similar AHR-dependent signaling events as B[a]P, PCB126 may interfere with the growth factor-triggered autophosphorylation of EGFR and downstream signal transduction required for *AKR1C3* induction.

### 3.6. The AHR- and EGFR-dependent upregulation of AKR1C3 is mediated by NRF2

**Fig. 4.**
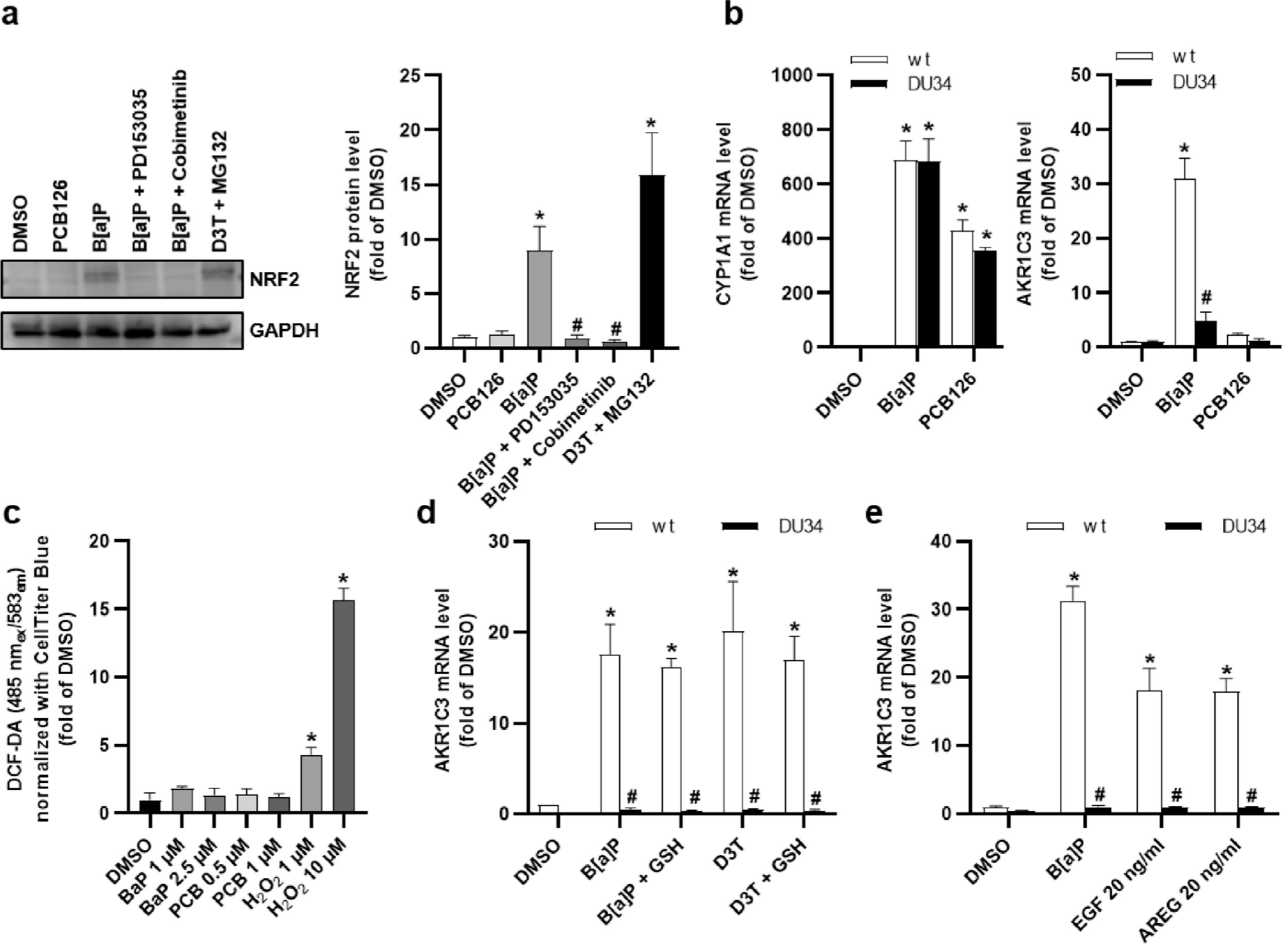
Induction of AKR1C3 depends on NRF2 in HaCaT keratinocytes. **a** Western blot analyses of NRF2 protein stabilization. Cells were treated as indicated, the co-treatment with D3T (70 μM) and MG-132 (10 μM) served as positive control. Whole cell lysates were prepared 6 h post stimulation. GAPDH was used as loading control. On the left a representative western blot, on the right the densitometric quantification is shown. n = 4. *, p ≤ 0.05 compared to DMSO, #, p ≤ 0.05 compared to B[a]P. **b** qRT-PCR analyses of *CYP1A1* and *AKR1C3* in HaCaT and HaCaT-NRF2-KO (DU34) keratinocytes. Cells were treated with B[a]P (2.5 μM), PCB126 (1 μM) or DMSO (0.1 %) DMSO for 24 h. n = 3. *, p ≤ 0.05 compared to DMSO HaCaT control, #, p ≤ 0.05 compared to the respective proficient HaCaT control. **c** ROS formation was analyzed using the DCF-DA assay. HaCaT cells were treated as indicated for 6 h. As a positive control cells were treated with H_2_O_2_ 30 min prior to staining. n = 3. *, p ≤ 0.05 compared to DMSO. **d** mRNA level of *AKR1C3* in HaCaT and HaCaT-NRF2-KO (DU34) cells treated for 24 h as indicated. Test compounds were used at the following concentrations: DMSO (0.1 %), B[a]P (2.5 μM), D3T (70 μM) and GSH (100 μM). n = 3. *, p ≤ 0.05 compared to DMSO, #, p ≤ 0.05 compared to the respective HaCaT control sample. **e** qRT-PCR analyses of *AKR1C3* in HaCaT and HaCaT-NRF2-KO (ΔNRF2) keratinocytes treated as indicated for 24 h. n = 4. *, p ≤ 0.05 compared to DMSO, #, p ≤ 0.05 compared to the respective HaCaT control sample.

Transcription factors of the AP-1 family are well-known executors of the MEK/ERK axis, a co-exposure of HaCaT cells to two pharmacological AP-1 inhibitors, however, did not reduce the B[a]P-mediated upregulation of *AKR1C3* (Fig. S5a). Another transcription factor known to control *AKR1C3* expression by binding to antioxidant response elements in its promoter sequence is NRF2 (Tebay et al., 2015). In unstressed cells, NRF2 is rapidly degraded by the proteasome. Upon oxidative, metabolic or oncogenic stress, NRF2 accumulates in the cytosol and subsequently translocates into the nucleus (Tebay et al., 2015). Interestingly, AHR stimulates NRF2 activity in ketoconazole-treated NHEKs independently of oxidative stress (Tsuji et al., 2012), and it was suggested that this process involves EGFR and downstream MEK/ERK signal transduction (Haarmann-Stemmann et al., 2012). We found that treatment of HaCaT keratinocytes for 6 h with either B[a]P or a combination of 1,2-dithiole-3-thione (D3T), which activates NRF2 *via* ERK (Manandhar et al., 2007), and the proteasome inhibitor MG132 resulted in an accumulation of NRF2 protein, whereas exposure to PCB126 did not (Fig. 4a). Accompanying qPCR analyses revealed a slight increase after 6 h and a significant increase of the AKR1C3 transcript numbers after 12 h of B[a]P treatment (Fig. S5b). Co-exposure to EGFR and MEK inhibitors attenuated the B[a]P-triggered accumulation of NRF2 (Fig. 4a), suggesting that NRF2 may be involved in the PAH-specific upregulation of AKR1C3. To confirm this, we next engineered NRF2-knockout HaCaT (HaCaT-NRF2-KO) cells, which neither expressed detectable amounts of NRF2 protein nor responded to a treatment with D3T by upregulating heme oxygenase-1 (*HO-1*) expression (Fig. S5c,d). Exposure of HaCaT and HaCaT-NRF2-KO cells to B[a]P and PCB126 resulted in an upregulation of *CYP1A1* in both cell-lines, whereas *AKR1C3* expression was only inducible by B[a]P in the NRF2-proficient keratinocytes (Fig. 4b). Depending on parameters, such as the capacity of the conjugating and antioxidant enzyme systems, an exposure to PAHs may cause the generation of reactive oxygen species (ROS). Interestingly, AKR1C3 is known to convert the CYP1A1-derived B[a]P metabolite B[a]P-7,8-*trans*-hydrodiol to B[a]P-7,8-catechol, which then may undergo redox-cycling (Park et al., 2008). To exclude this potential source of ROS, we generated CYP1A1-knockout HaCaT cells (HaCaT-CYP1A1-KO), which exhibited neither basal nor B[a]P-inducible CYP1A1 enzyme activity (Fig. S5e). As expected, exposure studies revealed a B[a]P- and PCB126-inducible expression of *CYP1A1* in the control cells, but not in the HaCaT-CYP1A1-KO cells. However, B[a]P treatment increased *AKR1C3* expression in both cell-lines (Fig. S5f) and thus independently from CYP1A1 activity and potentially associated ROS and genotoxic metabolites. This was confirmed by ROS measurements in HaCaT cells, showing no signs of oxidative stress 6 h after treatment with B[a]P and PCB126 (Fig. 4c). Moreover, neither the B[a]P- nor the D3T-induced upregulation of *AKR1C3* was significantly affected by a co-treatment with glutathione (Fig. 4d), which, as expected, efficiently decreased H_2_O_2_-related oxidative stress (Fig. S5g). In case the B[a]P-induced upregulation of *AKR1C3* occurred *via* the AHR-EGFR-NRF2 axis, a stimulation of HaCaT-NRF2-KO cells with EGFR ligands should not affect *AKR1C3* copy numbers. Indeed, exposure to both EGF and AREG induced *AKR1C3* expression to a similar extent in NRF2-proficient but not in NRF2-KO cells (Fig. 4e). Although we will not exclude an occurrence of ROS during the 24 h treatment with the AHR agonists, the above data argue against a major contribution of oxidative stress to the PAH-stimulated upregulation of AKR1C3. Taken together, our results strongly indicate that PAHs induce AKR1C3 expression in an AHR-,EGFR-, MEK/ERK- and NRF2-dependent manner and that PCB126 and related DLCs may disturb this signaling pathway at the level of EGFR.

### 3.7. DLCs bind to EGFR and inhibit its growth factor-induced activation

**Fig. 5.**
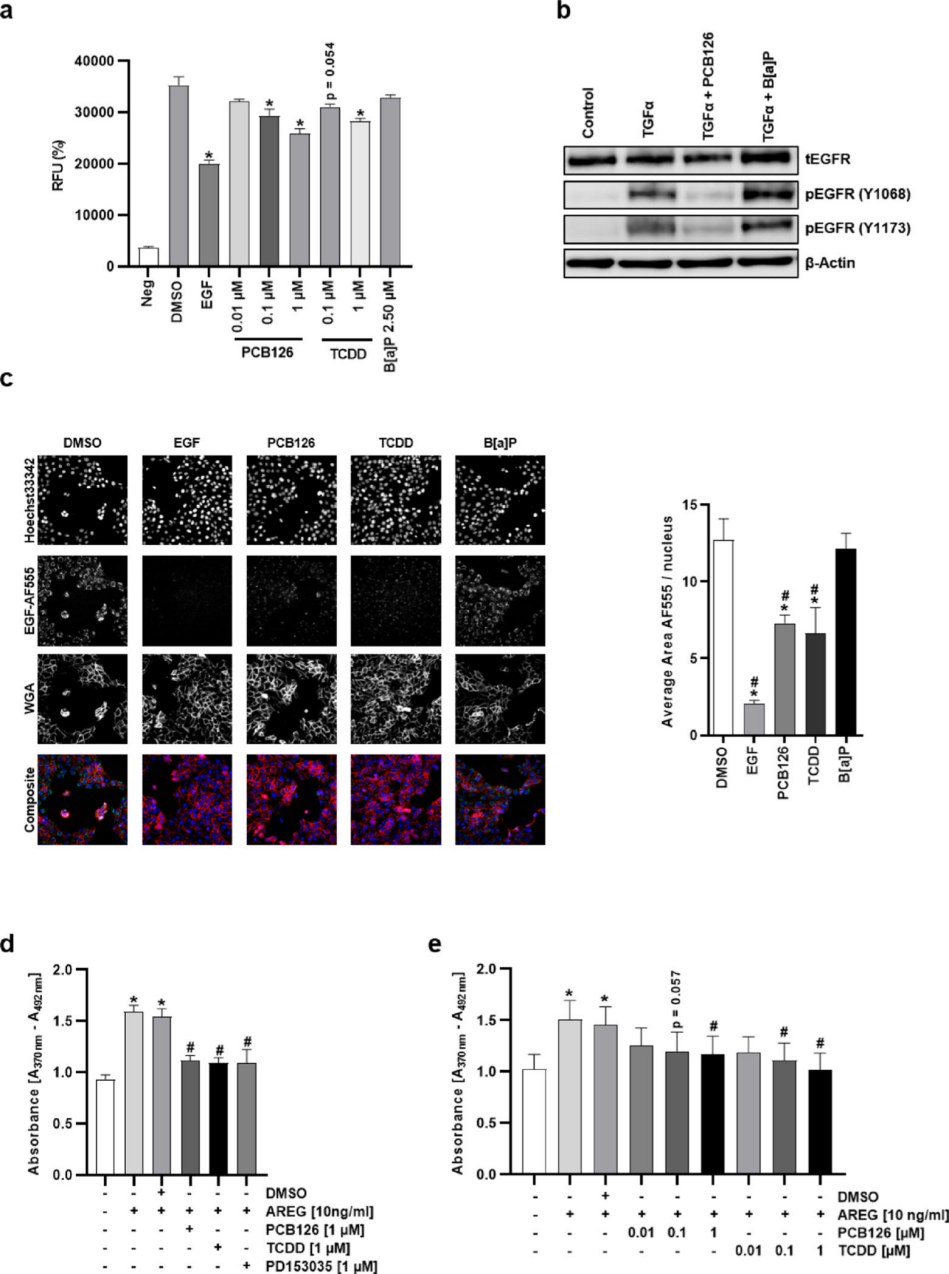
TCDD and PCB126 interfere with the activation and internalization of EGFR. **a** The effect of the DLCs on the interaction of EGF with EGFR was analyzed by using a cell-free EGF/EGFR AlphaLISA binding kit. n = 3. *, p ≤ 0.05 compared to DMSO. **b** Effect of B[a]P and PCB126 on EGFR activation upon TGFα stimulation was analyzed via western blot analyses. HaCaT keratinocytes were starved for 3 h and next stimulated with TGFα (20 ng/ml) for 2.5 min on ice. Afterwards, either DMSO (0.1 %), PCB126 (1 μM) or B[a]P (2.5 μM) was added and cells were incubated at 37 °C and 5 % CO_2_ for 30 min. Levels of total and phosphorylated EGFR (Y1068, Y1137) were determined; β-Actin was used as loading control. n = 3. representative pictures. **c** Influence of PCB126 (1 μM), TCDD (0.1 μM) and B[a]P (2.5 μM) on EGFR internalization was investigated by EGFR internalization assay and subsequent high content screening microscopy. On the left representative pictures, on the right results from the automated quantification are shown. n = 4–7. *, p ≤ 0.05 compared to DMSO. #, p ≤ 0.05 compared to B[a]P. **d** Colorimetric BrdU incorporation assay to assess the influence of PCB126, TCDD and PD153035 on AREG-induced DNA synthesis. HaCaT keratinocytes were treated as indicated for 4 h. Absorption was measured at a wavelength of 370 nm (reference wavelength 492 nm). n = 3. *, p ≤ 0.05 compared to DMSO. #, p ≤ 0.05 compared to AREG/DMSO. **e** Colorimetric BrdU incorporation assay in HaCaT-AHR-KO (DU26) keratinocytes. Cells were treated as indicated and the experiment was performed as described in **e**. n = 7. *, p ≤ 0.05 compared to DMSO. #, p ≤ 0.05 compared to AREG/DMSO.

The potential disturbance of the binding between EGFR and its ligands by DLCs was analyzed by using a cell-free AlphaLISA assay. The positive control, human recombinant EGF, reduced the binding of biotinylated EGF to antibody-captured EGFR by approximately 40% (Fig. 5a). As expected, the addition of B[a]P had no effect on ligand-receptor binding. PCB126, in contrast, inhibited the binding of biotinylated EGF to EGFR in a dose-dependent manner, and TCDD also slightly reduced ligand-receptor interaction (Fig. 5a). In addition, immunoblot analyses revealed that a 30 min co-treatment of HaCaT cells with PCB126, 2,3^′^,4,4^′^,5-pentachlorobiphenyl (PCB118), and TCDD nearly completely blunted the TGFα-induced phosphorylation of EGFR Y1068 and Y1173, while a treatment with B[a]P and B[k]F did not affect or even slightly increased it (Fig. 5b, Fig. S6a). We next performed high content analyses to monitor the effects of the DLCs on the internalization of the EGFR in HaCaT keratinocytes (Fig. 5c). A pretreatment of HaCaT cells with Alexa Fluor® 555-labeled EGF (AF-EGF) and a subsequent incubation with solvent (DMSO) led to an internalization of the AF-EGF-bound EGFR. An exposure of AF-EGF-pretreated cells with unlabeled EGF and increasing concentrations of PCB126 and TCDD dramatically reduced the internalization of EGFR, providing evidence that by binding to its ECD (Fig. 5c), DLCs interfere with the growth factor-induced activation of EGFR. To ensure that DLCs also inhibit functional endpoints of EGFR signaling, we assessed whether an exposure to PCB126 and TCDD affects EGFR ligand-stimulated DNA synthesis. Treatment of HaCaT keratinocytes with AREG induced DNA synthesis, which was inhibited by co-exposing the cells to either PCB126, TCDD or PD153035 (Fig. 5d). Subsequent dose response studies with TCDD, PCB118 and PCB126 in HaCaT-AHR-KO cells confirmed that DLCs inhibit AREG-induced DNA synthesis independently of AHR (Fig. 5e, Fig. S6b).

### 3.8. Docking analyses and mutational amino acid exchange identify EGFR residues required for DLC binding

**Fig. 6.**
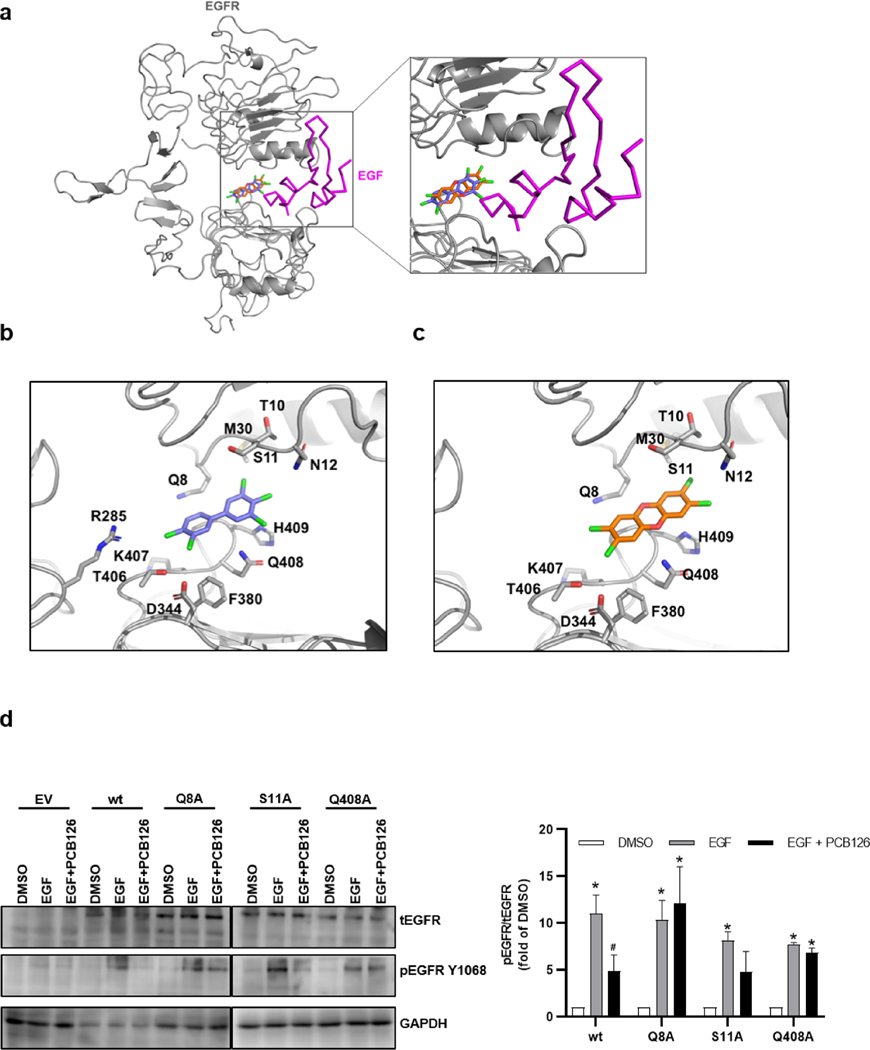
PCB126 and TCDD bind to EGFR and inhibit its growth factor-induced activation. **a** *In silico* docking analyses predicting the binding of PCB126 (blue) and TCDD (orange) to the ECD of EGFR (grey). EGF (magenta; taken from PDB ID: 1ivo) is superimposed. Interacting amino acid residues of EGFR ECD for **b** PCB126 and **c** TCDD shown as stick models. **d** Potentially interacting residues were converted to alanine by site directed mutagenesis. HepG2 cells were either transfected with an empty vector (pCMV3), pCMV3-EGFR plasmid or a plasmid bearing one of the following point mutations: EGFR^Q8A^, EGFR^S11A^ or EGFR^Q408A^. 24 h post transfection, cells were starved for 3 h and treated with EGF (10 ng/ml) alone and in combination with 1 μM PC126 for 15 min. Phosphorylation of EGFR residue Y1068 was assessed by western blotting and the results were normalized to total EGFR. GAPDH was used as loading control. Bar graph shows the densitometric quantification. Signals were compared with the respective DMSO control. n = 3. *, p ≤ 0.05 compared to DMSO, #, ≤ 0.05 compared to EGF. (For interpretation of the references to colour in this figure legend, the reader is referred to the web version of this article.)

To identify the molecular basis for the association between DLCs and EGFR, we conducted an *in silico* docking analysis for PCB126, TCDD and B[a]P with the ECD of human EGFR. In its inactive state the ECD adopts a “tethered” conformation that precludes EGFR dimerization. Binding of EGF or related growth factors stabilizes an extended ECD conformation, which can subsequently homo- or heterodimerize with another EGFR molecule or EGFR family member to build the active extended dimer (Chen et al., 2016). Our *in silico* docking analyses did not predict an interaction of any of the tested AHR ligands with the tethered EGFR monomer. Conversely, docking analyses with the extended ECD conformation predicted that TCDD and PCB126, but not B[a]P, bind to the same region on the ECD (domain I and III) of EGFR (Fig. 6a–c). The estimated binding energy of −11.6 kcal/mol (TCDD) and −11.1 kcal/ mol (PCB126) would correspond to dissociation constants of 55.7 nM and 59.3 nM, respectively (supplementary table 2). The site of interaction is next to the site where EGF intercalates to stabilize the extended ECD conformation (Fig. 6a). These analyses imply that binding of DLCs may stabilize the ECD in a slightly altered extended conformation that is incompatible with EGF binding and stable ECD dimerization. A further *in silico* docking analysis predicted that also other DLCs, namely PCB77, PCB118, PCDD, HCDD, and TCDF, are capable to interact with the EGFR ECD (Fig. S7a–f, supplementary table 2). Moreover, a superimposition of the compound structure of PCB126, TCDD and B[a]P (Fig. S7g) illustrated that due to its larger size, B[a]P probably clashes with EGFR residue Q8 and thus is unable to occupy the same binding site than DLCs (Fig. S7h).

To confirm the functional relevance of the amino acid residues predicted to be involved in DLC-binding (Fig. 6b,c, Fig. S7b–f, supplementary table 2), we mutated a cDNA expression plasmid to replace the amino acids at position 8 (glutamine), 11 (serine) and 408 (glutamine) of the mature EGFR protein by alanine. Transient transfection experiments in low EGFR-expressing human HepG2 hepatoma cells (Fuchs et al., 2008) revealed that the mutated EGFR variants (Q8A, S11A and Q408A) are still responsive towards EGF treatment, as indicated by an enhanced phosphorylation of EGFR at Y1068 (Fig. 6d). In co-exposure experiments with transfected cells expressing wild-type EGFR, PCB126 reduced the EGF-induced phosphorylation of the RTK. However, when we repeated this experiment in cells overexpressing the Q8A and Q408A EGFR variants, PCB126 did not affect the EGF-induced phosphorylation of EGFR Y1068 (Fig. 6d). These data provide compelling evidence that DLCs can directly bind to the ECD of EGFR to replace bound growth factors. Moreover, our data identify the EGFR residues Q8 and Q408 as being critical for the binding of DLCs to EGFR ECD.

### 3.9. EGFR inhibition shapes B[a]P-induced gene expression

**Fig. 7.**
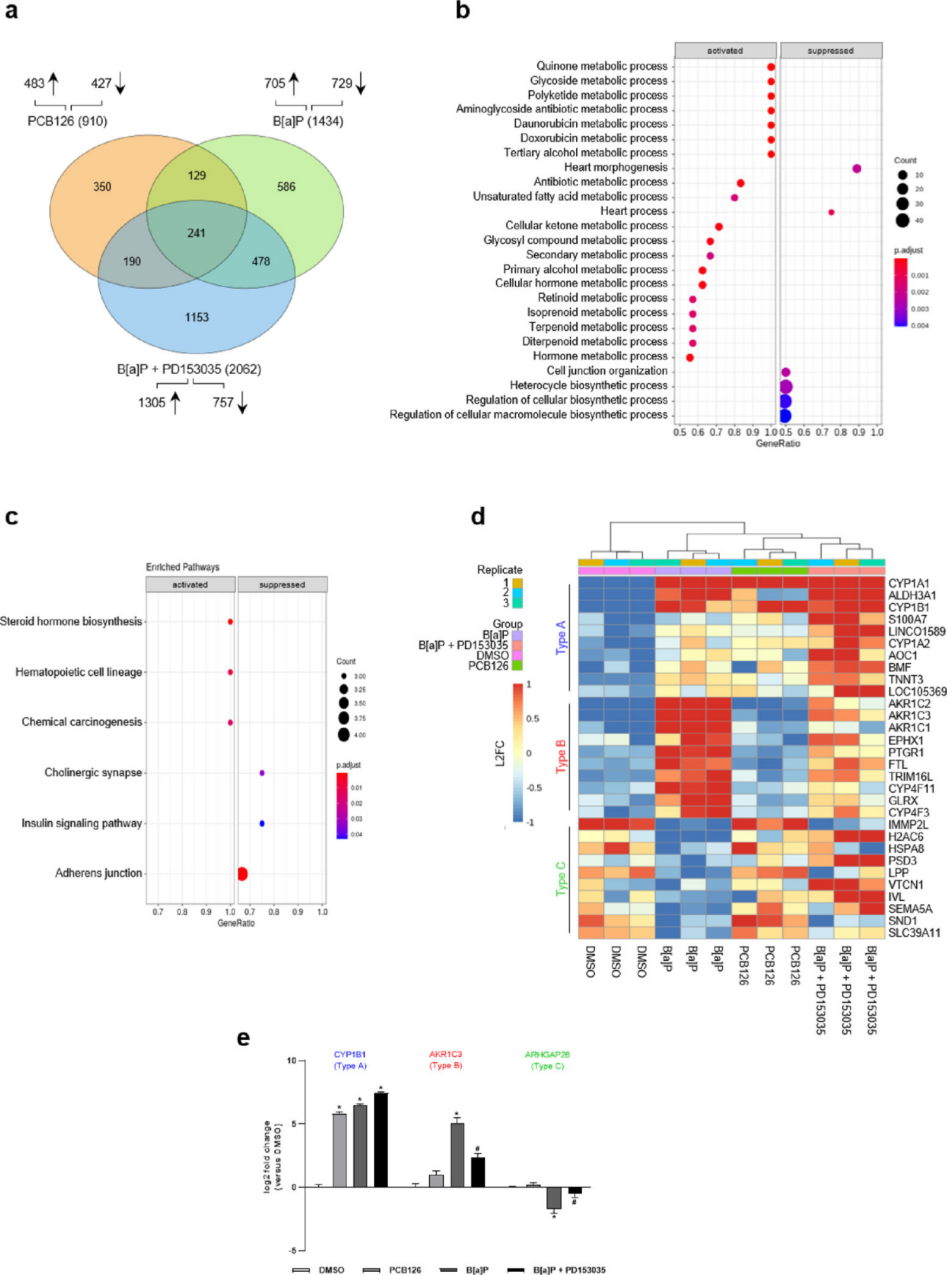
RNA-Seq analysis reveals distinct expression patterns upon B[a]P treatment. RNA-Seq analyses of HaCaT keratinocytes using oxford nanopore long-read RNA sequencing technology. Cells were treated with DMSO (0.1 %), PCB126 (1 μM), B[a]P (2.5 μM) or B[a]P (2.5 μM) + PD153035 (1 μM) for 24 h. n = 3. **a** Differentially expressed genes compared to DMSO with a |log_2_ fold change| ≥ 1.5 depicted in a venn diagram. **b** Gene set enrichment analysis of biological processes of a subset of filtered genes, which were solely regulated by B[a]P and where EGFR inhibition of EGFR signaling with PD153035 counteracted this regulation. Following filter strategy was applied: PCB126 vs. DMSO |log_2_ fold change| ≤ 1 → B[a]P vs PCB126 |log_2_ fold change| ≥ 2 → B[a]P vs B[a]P + PD153035 |log_2_ fold change| ≥ 1. Data set of differentially expression analysis of PCB126 vs. B[a]P was filtered for the remaining genes. Regulation of biological processes was depicted in a dot plot. **c** Genes filtered for **b** were employed on a KEGG pathway analysis. Regulated pathways are shown in a dot plot. **d** Variance heatmap of predefined expression patterns. Shown are the top 10 genes with the highest variance of each gene expression type. Type A: PCB126 vs. DMSO log_2_ fold change > 1.5 → B[a]P vs. DMSO log_2_ fold change > 1.5 → B[a]P + PD153035 vs. B[a]P log_2_ fold change > 0. Type B: PCB126 vs. DMSO log_2_ fold change < 1 → B[a]P vs. DMSO log_2_ fold change > 1.5 → B[a]P vs. B[a]P + PD153035 log_2_ fold change > 0. Type C: PCB126 vs. DMSO log_2_ fold change > 0 → B[a]P vs. DMSO log_2_ fold change < −1 → B[a]P vs. B[a]P + PD153035 log_2_ fold change < 0. **e** qRT-PCR analysis of genes representing the different types of gene expression patterns. n = 4. *, p ≤ 0.05 compared to DMSO, #, ≤ 0.05 compared to B[a]P.

To identify genes that are specifically regulated in response to B[a]P treatment and sensitive towards EGFR inhibition, we performed Nanopore long-read RNA sequencing of HaCaT cells treated for 24 h with PCB126, B[a]P alone and in combination with PD153035, and solvent (Fig. 7a). Compared to solvent control and considering a cut-off of | L2FC| ≥ 1.5, PCB126 and B[a]P treatment resulted in a differential expression of 910 and 1434 genes, respectively. A set of 370 genes was differentially regulated by both AHR agonists. In comparison to solvent control, the most molecular functions and pathways regulated by PCB126 or B[a]P treatment were related to xenobiotic metabolism, chemical carcinogenesis, steroid biosynthesis, and tryptophan metabolism (Fig. S8). Overall, suppression of gene expression, for instance related to cell adhesion and *O*-glycan biosynthesis, was more evident in response to B[a]P treatment (Fig. S8c,d). Moreover, 586 genes were solely regulated by B[a]P treatment. Given that these genes are not regulated in response to the combinatorial treatment, consisting of B[a] P and PD153035, they can be considered as being EGFR-dependent. In addition, the 478 genes representing the overlap between B[a]P- and B [a]P plus PD153035-treated samples, contains candidates whose expression is partially modulated in an EGFR-dependent manner (e.g. compared to DMSO, |L2FC| of cotreatment ≥ 1.5 but |L2FC| of cotreatment <|L2FC| B[a]P treatment). A gene set enrichment analysis revealed that the B[a]P-specific induction of various metabolic processes as well as the inhibition of biosynthetic pathways and cell junctional organization was sensitive towards pharmacological EGFR inhibition (Fig. 7b). KEGG pathway analysis indicated a B[a]P-induced and EGFR-dependent regulation of genes related to steroidogenesis, chemical carcinogenesis, insulin signaling and adherence junctions (Fig. 7c). Amongst others, genes regulated in response to B[a]P exposure can be categorized in three subtypes: ‘Type A’ represents genes that are upregulated *via* the canonical AHR pathway. The expression of these genes was induced upon PCB126 and B[a]P treatment and, because EGFR signaling dampens AHR/XRE-dependent gene expression (Sutter et al., 2009), further increased in cells co-treated with B[a]P and PD153035; ‘Type B’ represents genes that are positively regulated by non-canonical AHR signaling pathways. The expression of these genes was inducible by B[a]P but not by PCB126 treatment and sensitive towards EGFR inhibition; ‘Type C’ represents genes that are negatively regulated by non-canonical AHR signaling pathways. The expression of these genes was downregulated by B[a]P but not by PCB126 treatment and this downregulation was attenuated in response to PD153035 co-exposure. The top 10 regulated genes in terms of highest variance of each subtype are shown in the heatmap (Fig. 7d). The expression level of representative genes of each subtype was validated by qPCR (Fig. 7e, S9). Taken together, these results provide evidence that a substantial part of the B[a]P-induced alterations of the transcriptome is mediated by EGFR-dependent non-canonical AHR signaling pathways.

## 4. Discussion

Ligand-specificity of AHR signaling is multifactorial and influenced by pharmacokinetic aspects, compound-specific conformational changes of AHR and other parameters (Denison and Faber, 2017; Safe et al., 2020). By unraveling the specific responses induced by PAHs and DLCs, two classes of ubiquitous environmental chemicals comprising of multiple prototypic ligands, we here identified EGFR as a chemical-binding cell-surface receptor and critical determinant of AHR ligand specificity.

We provide evidence that PAHs induce EGFR signal transduction *via* two pathways, both initiated by an AHR ligand-driven stimulation of c-Src. Active c-Src rapidly phosphorylated EGFR (Y845) resulting in a phosphorylation of ERK. In addition, c-Src stimulated PKC and downstream metalloprotease activity to induce ectodomain shedding of cell surface-bound AREG and TGFα. These growth factors activated EGFR (Y1068, Y1173) in an auto-/paracrine fashion, resulting in a second peak of ERK phosphorylation and a NRF2-dependent induction of *AKR1C3*. On the contrary, PCB126 exposure, although enforcing PKC activation and growth factor shedding, neither caused a second peak of ERK activation nor an induction of *AKR1C3*.

It has been repeatedly reported that an exposure to AHR agonists interferes with the binding of radiolabeled EGF to the plasma membrane (Hudson et al., 1985; Kärenlampi et al., 1983; Madhukar et al., 1984). The underlying molecular mechanism of this interaction, however, is still enigmatic. It may be due to an AHR ligand-mediated enforcement of EGFR internalization either by stimulating a phosphorylation of the RTK *via* c-Src or by inducing the release of growth factors that bind to EGFR ECD. However, in contrast to PAHs that, presumably due to recycling of EGFR, only cause a transient decline (Hudson et al., 1985; Kärenlampi et al., 1983), TCDD reduces the EGF-binding capacity of the plasma membrane for up to 4 days (human keratinocytes) and 40 days (rat liver), respectively (Hudson et al., 1985; Madhukar et al., 1984). In light of our data, it is likely that DLCs interact with EGFR ECD and thereby disturb a proper binding of (labeled) EGF. This scenario would still allow a rapid AHR- and c-Src-dependent endogenous activation of EGFR (Y845) and downstream signal transduction by DLCs, but would exclude an activation of the RTK through auto-/paracrine events. In contrast to B [a]P, the DLCs tested in this study bind to the ECD of EGFR in its extended conformation in close proximity to the binding site for EGF. Our results are compatible with a model where DLC binding distorts the ECD sufficiently to block EGF binding and ECD dimerization. This model is supported by previous studies reporting an inhibition of growth factor-stimulated EGFR internalization by halogenated pesticides, PCBs and bisphenol S (Hardesty et al., 2018; Ticiani et al., 2021). The same mechanism as described for DLCs may thus be extended to other structurally related chemicals, making the EGFR a novel direct effector of environmental pollutants. However, given that several endogenous EGFR ligands are under transcriptional control of AHR (John et al., 2014; Patel et al., 2006), an accumulation of these growth factors over time may overcome the inhibitory effect on EGFR internalization of the ECD-bound DLCs.

In contrast to PCB126 and other DLCs, exposure of keratinocytes to B [a]P stimulates the growth factor-mediated activation of EGFR, thereby modulating the expression of hundreds of genes involved in various metabolic processes, biosynthetic pathways and cell adherence. DLCs in turn may specifically affect cellular functions by blocking growth factor-induced EGFR activation.

As mentioned above, a chronic exposure to PAHs may foster the onset and worsening of atopic diseases, whereas an exposure to DLCs has no or even the opposite effect on the pathogenesis of these Th2-driven diseases. The differential regulation of PGD_2_ metabolism may partially contribute to this AHR ligand-specific difference. PGD_2_ is mainly released by degranulating mast cells and contributes to the pathogenesis of allergic inflammatory diseases by binding to CRTH2 on Th2 cells and stimulating cytokine secretion (Pettipher et al., 2007). However, upon its release PGD_2_ either spontaneously hydrolyzes to 15-deoxyΔ12–14 PGJ_2_ or is reduced by AKR1C3 to 9α,11β-PGF_2_. Whereas 15-deoxyΔ12–14 PGJ_2_ acts as an anti-inflammatory (Pettipher et al., 2007), 9α,11β-PGF_2_, which is less potent in activating CRTH2 than PGD_2_ but metabolically stable, prolongs allergic inflammation and serves as systemic biomarker for allergen-induced mast cell activation (Bochenek et al., 2003; Seibert et al., 1987). In the skin, elevated expression levels of AKR1C3 and the associated formation of 9α,11β-PGF_2_ may stimulate a Th2 cytokine-induced disturbance of keratinocyte differentiation (Mantel et al., 2012). In addition, by converting PAH diols to redox-cycling catechols, enhanced AKR1C3 levels may enforce mast cell activation and cause further oxidative tissue damage (Diaz-Sanchez et al., 2000; Park et al., 2008). A PAH-driven activation of AHR may thus contribute to allergic inflammatory diseases by perpetuating Th2 responses, impairing epithelial barrier function and enhancing the susceptibility towards inflammatory stimuli (Dijkhoff et al., 2020; Lag et al., 2020). For regulatory purposes, it should be considered that the mouse genome encodes neither AKR1C3 nor a functional homolog (Velica et al., 2009).

Regarding its important role in development and tissue homeostasis (Chen et al., 2016), an inhibition of growth factor-driven EGFR activation may be a critical mode of action of DLCs. In the skin, for instance, EGFR signaling contributes to epidermal homeostasis by tightly orchestrating keratinocyte fate and associated barrier functions (Chen et al., 2016). A systemic application of EGFR inhibitors, e.g. during lung cancer therapy, is frequently accompanied by cutaneous adverse effects, including an acceleration of keratinocyte differentiation and an impairment of the epidermal barrier (Lacouture, 2006; Lichtenberger et al., 2013). Accordingly, treatment of human keratinocytes with EGFR inhibitors switches the keratinocyte program from proliferation to differentiation (Lichtenberger et al., 2013; Peus et al., 1997). This program switch is also observed in human keratinocytes exposed to TCDD and may contribute to the pathogenesis of chloracne, the hallmark of an acute intoxication with DLCs in humans (Hudson et al., 1985; Sutter et al., 2019).

From a mechanistical point of view, a chemical inhibition of growth factor-driven proliferation and an induction of cellular differentiation may be of interest for the treatment of EGFR-positive cancers. In fact, results from rodent studies revealed an anti-carcinogenic effect of TCDD in certain tissues (DiGiovanni et al., 1980; Holcomb and Safe, 1994). However, whether DLCs may serve as a structural blueprint for the development of novel non-toxic EGFR ECD modulators for therapeutic purposes remains to be investigated. A limitation of this study is that we have not yet confirmed a direct interaction of DLCs with EGFR molecules from other species than human. However, a multiple alignment of the *N*-terminal 420 amino acids of the mature EGFR protein revealed a high conservation of the residues predicted and/or experimentally proven to be involved in DLC binding across several mammalian species (Fig. S10).

Taken together, we identified a PAH-specific non-canonical AHR signaling pathway responsible for the induction of AKR1C3 and the associated 11-ketoreduction of PGD_2_ that provides a plausible link between PAH exposure and the pathogenesis of allergic inflammatory diseases. In addition, we revealed that DLCs interact with EGFR ECD, resulting in a displacement of bound growth factors and an inhibition of downstream events. This novel mode of action is probably relevant for various DLC-associated adverse health effects, in particular for those involving a dysbalance between cell proliferation and differentiation. We conclude that the expression level of EGFR and the presence of its ligands are critical parameters which have to be considered in order to optimize the prediction of the biological outcome in a given PAH- or DLC-exposed cell population or tissue.

## Supplementary Material

word doc

spread sheet

## References

[R1] Bessede A, Gargaro M, Pallotta MT, Matino D, Servillo G, Brunacci C, Bicciato S, Mazza EMC, Macchiarulo A, Vacca C, Iannitti R, Tissi L, Volpi C, Belladonna ML, Orabona C, Bianchi R, Lanz TV, Platten M, DellaFazia MA, Piobbico D, Zelante T, Funakoshi H, Nakamura T, Gilot D, Denison MS, Guillemin GJ, DuHadaway JB, Prendergast GC, Metz R, Geffard M, Boon L, Pirro M, Iorio A, Veyret B, Romani L, Grohmann U, Fallarino F, Puccetti P, 2014. Aryl hydrocarbon receptor control of a disease tolerance defence pathway. Nature 511 (7508), 184–190. 24930766 10.1038/nature13323 PMC4098076

[R2] Bochenek G, Nagraba K, Nizankowska E, Szczeklik A, 2003. A controlled study of 9alpha,11beta-PGF2 (a prostaglandin D2 metabolite) in plasma and urine of patients with bronchial asthma and healthy controls after aspirin challenge. J. Allergy Clin. Immunol 111, 743–749. 12704352 10.1067/mai.2003.1387

[R3] Burczynski ME, Lin HK, Penning TM, 1999. Isoform-specific induction of a human aldo-keto reductase by polycyclic aromatic hydrocarbons (PAHs), electrophiles, and oxidative stress: implications for the alternative pathway of PAH activation catalyzed by human dihydrodiol dehydrogenase. Cancer Res. 59, 607–614. 9973208

[R4] Castañeda AR, Vogel CFA, Bein KJ, Hughes HK, Smiley-Jewell S, Pinkerton KE, 2018. Ambient particulate matter enhances the pulmonary allergic immune response to house dust mite in a BALB/c mouse model by augmenting Th2- and Th17-immune responses. Physiol. Rep 6 (18), e13827. 10.14814/phy2.13827. 30230272 PMC6144457

[R5] Chen J, Zeng F, Forrester SJ, Eguchi S, Zhang M-Z, Harris RC, 2016. Expression and Function of the Epidermal Growth Factor Receptor in Physiology and Disease. Physiol. Rev 96 (3), 1025–1069. 33003261 10.1152/physrev.00030.2015

[R6] Denison MS, Faber SC, 2017. And now for something completely different: diversity in ligand-dependent activation of Ah receptor responses. Curr. Opin. Toxicol 2, 124–131. 28845473 10.1016/j.cotox.2017.01.006 PMC5570615

[R7] Diaz-Sanchez D, Penichet-Garcia M, Saxon A, 2000. Diesel exhaust particles directly induce activated mast cells to degranulate and increase histamine levels and symptom severity. J. Allergy Clin. Immunol 106 (6), 1140–1146. 11112898 10.1067/mai.2000.111144

[R8] DiGiovanni J, Berry DL, Gleason GL, Kishore GS, Slaga TJ, 1980. Time-dependent inhibition by 2,3,7,8-tetrachlorodibenzo-p-dioxin of skin tumorigenesis with polycyclic hydrocarbons. Cancer Res. 40, 1580–1587. 6768449

[R9] Dijkhoff IM, Drasler B, Karakocak BB, Petri-Fink A, Valacchi G, Eeman M, Rothen-Rutishauser B, 2020. Impact of airborne particulate matter on skin: a systematic review from epidemiology to in vitro studies. Part. Fibre Toxicol 17, 35. 32711561 10.1186/s12989-020-00366-y PMC7382801

[R10] Dong B, Cheng W, Li W, Zheng J, Wu D, Matsumura F, Vogel CFA, 2011. FRET analysis of protein tyrosine kinase c-Src activation mediated via aryl hydrocarbon receptor. BBA 1810 (4), 427–431. 21145940 10.1016/j.bbagen.2010.11.007 PMC3049970

[R11] Frauenstein K, Tigges J, Soshilov AA, Kado S, Raab N, Fritsche E, Haendeler J, Denison MS, Vogel CFA, Haarmann-Stemmann T, 2015. Activation of the arylhydrocarbon receptor by the widely used Src family kinase inhibitor 4-amino-5-(4-chlorophenyl)-7-(dimethylethyl)pyrazolo[3,4-d]pyrimidine (PP2). Arch. Toxicol 89 (8), 1329–1336. 25082669 10.1007/s00204-014-1321-8 PMC4454626

[R12] Fritsche E, Schafer C, Calles C, Bernsmann T, Bernshausen T, Wurm M, Hubenthal U, Cline JE, Hajimiragha H, Schroeder P, Klotz L-O, Rannug A, Furst P, Hanenberg H, Abel J, Krutmann J, 2007. Lightening up the UV response by identification of the arylhydrocarbon receptor as a cytoplasmatic target for ultraviolet B radiation. PNAS 104 (21), 8851–8856. 17502624 10.1073/pnas.0701764104 PMC1885591

[R13] Fuchs BC, Fujii T, Dorfman JD, Goodwin JM, Zhu AX, Lanuti M, Tanabe KK, 2008. Epithelial-to-mesenchymal transition and integrin-linked kinase mediate sensitivity to epidermal growth factor receptor inhibition in human hepatoma cells. Cancer Res. 68 (7), 2391–2399. 18381447 10.1158/0008-5472.CAN-07-2460

[R14] Funatake CJ, Marshall NB, Steppan LB, Mourich DV, Kerkvliet NI, 2005. Cutting edge: activation of the aryl hydrocarbon receptor by 2,3,7,8-tetrachlorodibenzo-p-dioxin generates a population of CD4+CD25+cells with characteristics of regulatory T cells. J. Immunol 175 (7), 4184–4188. 16177056 10.4049/jimmunol.175.7.4184

[R15] Goodsell DS, Morris GM, Olson AJ, 1996. Automated docking of flexible ligands: applications of AutoDock. J. Mol. Recognit 9 (1), 1–5. 8723313 10.1002/(sici)1099-1352(199601)9:1<1::aid-jmr241>3.0.co;2-6

[R16] Haarmann-Stemmann T, Abel J, Fritsche E, Krutmann J, 2012. The AhR-Nrf2pathway in keratinocytes: on the road to chemoprevention? J, Invest. Dermatol 132 (1), 7–9. 22158605 10.1038/jid.2011.359

[R17] Hardesty JE, Al-Eryani L, Wahlang B, Falkner KC, Shi H, Jin J, Vivace BJ, Ceresa BP, Prough RA, Cave MC, 2018. Epidermal Growth Factor Receptor Signaling Disruption by Endocrine and Metabolic Disrupting Chemicals. Toxicol. Sci 162, 622–634. 29329451 10.1093/toxsci/kfy004 PMC5888991

[R18] Hawerkamp HC, Kislat A, Gerber PA, Pollet M, Rolfes KM, Soshilov AA, Denison MS, Momin AA, Arold ST, Datsi A, Braun SA, Oláh P, Lacouture ME, Krutmann J, Haarmann-Stemmann T, Homey B, Meller S, 2019. Vemurafenib acts as an aryl hydrocarbon receptor antagonist: Implications for inflammatory cutaneous adverse events. Allergy 74 (12), 2437–2448. 31269229 10.1111/all.13972 PMC6911016

[R19] Hidaka T, Ogawa E, Kobayashi EH, Suzuki T, Funayama R, Nagashima T, Fujimura T, Aiba S, Nakayama K, Okuyama R, Yamamoto M, 2017. The arylhydrocarbon receptor AhR links atopic dermatitis and air pollution via induction of the neurotrophic factor artemin. Nat. Immunol 18 (1), 64–73. 27869817 10.1038/ni.3614

[R20] Holcomb M, Safe S, 1994. Inhibition of 7,12-dimethylbenzanthracene-induced rat mammary tumor growth by 2,3,7,8-tetrachlorodibenzo-p-dioxin. Cancer Lett. 82 (1), 43–47. 8033067 10.1016/0304-3835(94)90144-9

[R21] Hong C-H, Lee C-H, Yu H-S, Huang S-K, 2016. Benzopyrene, a major polyaromatic hydrocarbon in smoke fume, mobilizes Langerhans cells and polarizes Th2/17 responses in epicutaneous protein sensitization through the arylhydrocarbon receptor. Int. Immunopharmacol 36, 111–117. 27129092 10.1016/j.intimp.2016.04.017

[R22] Hudson LG, Toscano WA, Greenlee WF, 1985. Regulation of epidermal growth factor binding in a human keratinocyte cell line by 2,3,7,8-tetrachlorodibenzo-p-dioxin. Toxicol. Appl. Pharmacol 77 (2), 251–259. 2579474 10.1016/0041-008x(85)90324-2

[R23] John K, Lahoti TS, Wagner K, Hughes JM, Perdew GH, 2014. The Ah receptor regulates growth factor expression in head and neck squamous cell carcinoma cell lines. Mol. Carcinog 53 (10), 765–776. 23625689 10.1002/mc.22032 PMC4388041

[R24] Joseloff E, Cataisson C, Aamodt H, Ocheni H, Blumberg P, Kraker AJ, Yuspa SH, 2002. Src family kinases phosphorylate protein kinase C delta on tyrosine residues and modify the neoplastic phenotype of skin keratinocytes. J. Biol. Chem 277, 12318–12323. 11812791 10.1074/jbc.M111618200

[R25] Kärenlampi SO, Eisen HJ, Hankinson O, Nebert DW, 1983. Effects of cytochrome P1–450 inducers on the cell-surface receptors for epidermal growth factor, phorbol 12,13-dibutyrate, or insulin of cultured mouse hepatoma cells. J. Biol. Chem 258 (17), 10378–10383. 6309801

[R26] Kim S, Thiessen PA, Bolton EE, Chen J, Fu G, Gindulyte A, Han L, He J, He S, Shoemaker BA, Wang J, Yu B.o., Zhang J, Bryant SH, 2016. PubChem Substance and Compound databases. Nucleic Acids Res. 44 (D1), D1202–D1213. 26400175 10.1093/nar/gkv951 PMC4702940

[R27] Kopec AK, Burgoon LD, Ibrahim-Aibo D, Burg AR, Lee AW, Tashiro C, Potter D, Sharratt B, Harkema JR, Rowlands JC, Budinsky RA, Zacharewski TR, 2010. Automated dose-response analysis and comparative toxicogenomic evaluation of the hepatic effects elicited by TCDD, TCDF, and PCB126 in C57BL/6 mice. Toxicol. Sci 118, 286–297. 20702594 10.1093/toxsci/kfq236 PMC2955213

[R28] Lacouture ME, 2006. Mechanisms of cutaneous toxicities to EGFR inhibitors. Nat. Rev. Cancer 6 (10), 803–812. 16990857 10.1038/nrc1970

[R29] Lag M, Ovrevik J, Refsnes M, Holme JA, 2020. Potential role of polycyclic aromatic hydrocarbons in air pollution-induced non-malignant respiratory diseases. Respir. Res 21, 299. 33187512 10.1186/s12931-020-01563-1 PMC7666487

[R30] Li H, 2018. Minimap2: pairwise alignment for nucleotide sequences. Bioinformatics 34, 3094–3100. 29750242 10.1093/bioinformatics/bty191 PMC6137996

[R31] Liao Y, Smyth GK, Shi W, 2019. The R package Rsubread is easier, faster, cheaper and better for alignment and quantification of RNA sequencing reads. Nucleic Acids Res. 47 e47. 30783653 10.1093/nar/gkz114 PMC6486549

[R32] Lichtenberger BM, Gerber PA, Holcmann M, Buhren BA, Amberg N, Smolle V, Schrumpf H, Boelke E, Ansari P, Mackenzie C, Wollenberg A, Kislat A, Fischer JW, Rock K, Harder J, Schroder JM, Homey B, Sibilia M, 2013. Epidermal EGFR controls cutaneous host defense and prevents inflammation. Sci. Transl. Med 5, 199ra111. 10.1126/scitranslmed.3005886 23966300

[R33] Liu Y, He S, Chen Y, Liu Y, Feng F, Liu W, Guo Q, Zhao L.i., Sun H, 2020. Overview of AKR1C3: Inhibitor Achievements and Disease Insights. J. Med. Chem 63 (20), 11305–11329. 32463235 10.1021/acs.jmedchem.9b02138

[R34] Love MI, Huber W, Anders S, 2014. Moderated estimation of fold change and dispersion for RNA-seq data with DESeq2. Genome Biol. 15, 550. 25516281 10.1186/s13059-014-0550-8 PMC4302049

[R35] Madhukar BV, Brewster DW, Matsumura F, 1984. Effects of in vivo-administered 2,3,7,8-tetrachlorodibenzo-p-dioxin on receptor binding of epidermal growth factor in the hepatic plasma membrane of rat, guinea pig, mouse, and hamster. PNAS 81 (23), 7407–7411. 6095293 10.1073/pnas.81.23.7407 PMC392155

[R36] Manandhar S, Cho J-M, Kim J-A, Kensler TW, Kwak M-K, 2007. Induction of Nrf2-regulated genes by 3H–1, 2-dithiole-3-thione through the ERK signaling pathway in murine keratinocytes. Eur. J. Pharmacol 577 (1–3), 17–27. 17854798 10.1016/j.ejphar.2007.08.018

[R37] Mantel A, Carpenter-Mendini AB, VanBuskirk JB, De Benedetto A, Beck LA, Pentland AP, 2012. Aldo-keto reductase 1C3 is expressed in differentiated human epidermis, affects keratinocyte differentiation, and is upregulated in atopic dermatitis. J, Invest. Dermatol 132 (4), 1103–1110. 22170488 10.1038/jid.2011.412 PMC3305848

[R38] Murray IA, Patterson AD, Perdew GH, 2014. Aryl hydrocarbon receptor ligands in cancer: friend and foe. Nat. Rev. Cancer 14 (12), 801–814. 25568920 10.1038/nrc3846 PMC4401080

[R39] Nadeau K, McDonald-Hyman C, Noth EM, Pratt B, Hammond SK, Balmes J, Tager I, 2010. Ambient air pollution impairs regulatory T-cell function in asthma. J. Allergy Clin. Immunol 126 (4), 845–852.e10. 20920773 10.1016/j.jaci.2010.08.008

[R40] Nakamoto M, Arisawa K, Uemura H, Katsuura S, Takami H, Sawachika F, Yamaguchi M, Juta T, Sakai T, Toda E, Mori K, Hasegawa M, Tanto M, Shima M, Sumiyoshi Y, Morinaga K, Kodama K, Suzuki T, Nagai M, Satoh H, 2013. Association between blood levels of PCDDs/PCDFs/dioxin-like PCBs and history of allergic and other diseases in the Japanese population. Int. Arch. Occup. Environ. Health 86 (8), 849–859. 23014754 10.1007/s00420-012-0819-8

[R41] Nault R, Forgacs AL, Dere E, Zacharewski TR, 2013. Comparisons of differential gene expression elicited by TCDD, PCB126, betaNF, or ICZ in mouse hepatoma Hepa1c1c7 cells and C57BL/6 mouse liver. Toxicol. Lett 223, 52–59. 23994337 10.1016/j.toxlet.2013.08.013 PMC4096832

[R42] O’Boyle NM, Banck M, James CA, Morley C, Vandermeersch T, Hutchison GR, 2011. Open Babel: An open chemical toolbox. J. Cheminform 3, 33. 21982300 10.1186/1758-2946-3-33 PMC3198950

[R43] O’Driscoll CA, Owens LA, Gallo ME, Hoffmann EJ, Afrazi A, Han M, Fechner JH, Schauer JJ, Bradfield CA, Mezrich JD, 2018. Differential effects of diesel exhaust particles on T cell differentiation and autoimmune disease. Part. Fibre Toxicol 15, 35. 30143013 10.1186/s12989-018-0271-3 PMC6109291

[R44] Park J-H, Mangal D, Tacka KA, Quinn AM, Harvey RG, Blair IA, Penning TM, 2008. Evidence for the aldo-keto reductase pathway of polycyclic aromatic trans-dihydrodiol activation in human lung A549 cells. Proc. Natl. Acad. Sci. USA 105 (19), 6846–6851. 18474869 10.1073/pnas.0802776105 PMC2383938

[R45] Patel RD, Kim DJ, Peters JM, Perdew GH, 2006. The aryl hydrocarbon receptor directly regulates expression of the potent mitogen epiregulin. Toxicol. Sci 89, 75–82. 16192470 10.1093/toxsci/kfi344

[R46] Penning TM, 2019. AKR1C3 (type 5 17beta-hydroxysteroid dehydrogenase/prostaglandin F synthase): Roles in malignancy and endocrine disorders. Mol. Cell. Endocrinol 489, 82–91. 30012349 10.1016/j.mce.2018.07.002 PMC6422768

[R47] Pettipher R, Hansel TT, Armer R, 2007. Antagonism of the prostaglandin D2 receptors DP1 and CRTH2 as an approach to treat allergic diseases. Nat. Rev. Drug Discov 6 (4), 313–325. 17396136 10.1038/nrd2266

[R48] Peus D, Hamacher L, Pittelkow MR, 1997. EGF-receptor tyrosine kinase inhibition induces keratinocyte growth arrest and terminal differentiation. J. Invest. Dermatol 109 (6), 751–756. 9406816 10.1111/1523-1747.ep12340759

[R49] Quintana FJ, Basso AS, Iglesias AH, Korn T, Farez MF, Bettelli E, Caccamo M, Oukka M, Weiner HL, 2008. Control of T(reg) and T(H)17 cell differentiation by the aryl hydrocarbon receptor. Nature 453 (7191), 65–71. 18362915 10.1038/nature06880

[R50] Ramachandran H, Martins S, Kontarakis Z, Krutmann J, Rossi A, 2021. Fast but not furious: a streamlined selection method for genome-edited cells. Life Sci Alliance 4. 10.26508/lsa.202101051 PMC8127327 33903218

[R51] Roman ÁC, Carvajal-Gonzalez JM, Merino JM, Mulero-Navarro S, Fernández-Salguero PM, 2018. The aryl hydrocarbon receptor in the crossroad of signalling networks with therapeutic value. Pharmacol. Ther 185, 50–63. 29258844 10.1016/j.pharmthera.2017.12.003

[R52] Rothhammer V, Quintana FJ, 2019. The aryl hydrocarbon receptor: an environmental sensor integrating immune responses in health and disease. Nat. Rev. Immunol 19 (3), 184–197. 30718831 10.1038/s41577-019-0125-8

[R53] Safe S, Jin U. h., Park H, Chapkin RS, Jayaraman A, 2020. Aryl Hydrocarbon Receptor (AHR) Ligands as Selective AHR Modulators (SAhRMs). Int. J. Mol. Sci 21 (18), 6654. 10.3390/ijms21186654. PMC7555580 32932962

[R54] Seals DF, Courtneidge SA, 2003. The ADAMs family of metalloproteases: multidomain proteins with multiple functions. Genes Dev. 17 (1), 7–30. 12514095 10.1101/gad.1039703

[R55] Seibert K, Sheller JR, Roberts LJ, 1987. (5Z,13E)-(15S)-9 alpha,11 beta,15-trihydroxyprosta-5,13-dien-1-oic acid (9 alpha,11 beta-prostaglandin F2): formation and metabolism by human lung and contractile effects on human bronchial smooth muscle. PNAS 84 (1), 256–260. 3467352 10.1073/pnas.84.1.256 PMC304182

[R56] Sievers F, Wilm A, Dineen D, Gibson TJ, Karplus K, Li W, Lopez R, McWilliam H, Remmert M, Söding J, Thompson JD, Higgins DG, 2011. Fast, scalable generation of high-quality protein multiple sequence alignments using Clustal Omega. Mol. Syst. Biol 7 (1), 539. 10.1038/msb:2011.75. 21988835 PMC3261699

[R57] Souza T, Jennen D, van Delft J, van Herwijnen M, Kyrtoupolos S, Kleinjans J, 2016. New insights into BaP-induced toxicity: role of major metabolites in transcriptomics and contribution to hepatocarcinogenesis. Arch. Toxicol 90 (6), 1449–1458. 26238291 10.1007/s00204-015-1572-z PMC4873527

[R58] Stockinger B, Shah K, Wincent E, 2021. AHR in the intestinal microenvironment: safeguarding barrier function. Nat. Rev. Gastroenterol. Hepatol 18 (8), 559–570. 33742166 10.1038/s41575-021-00430-8 PMC7611426

[R59] Sutter CH, Olesen KM, Bhuju J, Guo Z, Sutter TR, 2019. AHR Regulates Metabolic Reprogramming to Promote SIRT1-Dependent Keratinocyte Differentiation. J. Invest. Dermatol 139 (4), 818–826. 30393078 10.1016/j.jid.2018.10.019 PMC6431567

[R60] Sutter CH, Yin H, Li Y, Mammen JS, Bodreddigari S, Stevens G, Cole JA, Sutter TR, 2009. EGF receptor signaling blocks aryl hydrocarbon receptor-mediated transcription and cell differentiation in human epidermal keratinocytes. Proc. Natl. Acad. Sci. USA 106 (11), 4266–4271. 19255421 10.1073/pnas.0900874106 PMC2649958

[R61] Tebay LE, Robertson H, Durant ST, Vitale SR, Penning TM, Dinkova-Kostova AT, Hayes JD, 2015. Mechanisms of activation of the transcription factor Nrf2 by redox stressors, nutrient cues, and energy status and the pathways through which it attenuates degenerative disease. Free Radic. Biol. Med 88, 108–146. 26122708 10.1016/j.freeradbiomed.2015.06.021 PMC4659505

[R62] Ticiani E, Gingrich J, Pu Y, Vettathu M, Davis J, Martin D, Petroff MG, Veiga-Lopez A, 2021. Bisphenol S and Epidermal Growth Factor Receptor Signaling in Human Placental Cytotrophoblasts. Environ. Health Perspect 129 (2), 027005. 10.1289/EHP7297. PMC7894408 33605785

[R63] Tsuji G, Takahara M, Uchi H, Matsuda T, Chiba T, Takeuchi S, Yasukawa F, Moroi Y, Furue M, 2012. Identification of ketoconazole as an AhR-Nrf2 activator in cultured human keratinocytes: the basis of its anti-inflammatory effect. J, Invest. Dermatol 132 (1), 59–68. 21753779 10.1038/jid.2011.194

[R64] Velica P, Davies NJ, Rocha PP, Schrewe H, Ride JP, Bunce CM, 2009. Lack of functional and expression homology between human and mouse aldo-keto reductase 1C enzymes: implications for modelling human cancers. Mol. Cancer 8, 121. 20003443 10.1186/1476-4598-8-121 PMC2805611

[R65] Vogel CFA, Lazennec G, Kado SY, Dahlem C, He Y, Castaneda A, Ishihara Y, Vogeley C, Rossi A, Haarmann-Stemmann T, Jugan J, Mori H, Borowsky AD, La Merrill MA, Sweeney C, 2021. Targeting the Aryl Hydrocarbon Receptor Signaling Pathway in Breast Cancer Development. Front. Immunol 12, 625346. 33763068 10.3389/fimmu.2021.625346 PMC7982668

[R66] Vogel CFA, Van Winkle LS, Esser C, Haarmann-Stemmann T, 2020. The aryl hydrocarbon receptor as a target of environmental stressors - Implications for pollution mediated stress and inflammatory responses. Redox Biol. 34, 101530. 10.1016/j.redox.2020.101530. 32354640 PMC7327980

[R67] Wang J, Xie X, 2007. Development of a quantitative, cell-based, high-content screening assay for epidermal growth factor receptor modulators. Acta Pharmacol. Sin 28 (10), 1698–1704. 17883960 10.1111/j.1745-7254.2007.00640.x

[R68] Weng C-M, Wang C-H, Lee M-J, He J-R, Huang H-Y, Chao M-W, Chung KF, Kuo H-P, 2018. Aryl hydrocarbon receptor activation by diesel exhaust particles mediates epithelium-derived cytokines expression in severe allergic asthma. Allergy 73 (11), 2192–2204. 29672862 10.1111/all.13462

[R69] Wong TH, Lee CL, Su HH, Lee CL, Wu CC, Wang CC, Sheu CC, Lai RS, Leung SY, Lin CC, Wei YF, Wang CJ, Lin YC, Chen HL, Huang MS, Yen JH, Huang SK, Suen JL, 2018. A prominent air pollutant, Indeno[1,2,3-cd]pyrene, enhances allergic lung inflammation via aryl hydrocarbon receptor. Sci. Rep 8, 5198. 29581487 10.1038/s41598-018-23542-9 PMC5979946

[R70] Xia M, Viera-Hutchins L, Garcia-Lloret M, Noval Rivas M, Wise P, McGhee SA, Chatila ZK, Daher N, Sioutas C, Chatila TA, 2015. Vehicular exhaust particles promote allergic airway inflammation through an aryl hydrocarbon receptor-notch signaling cascade. J. Allergy Clin. Immunol 136 (2), 441–453. 25825216 10.1016/j.jaci.2015.02.014 PMC4530027

[R71] Ye M, Warner M, Mocarelli P, Brambilla P, Eskenazi B, 2018. Prenatal exposure to TCDD and atopic conditions in the Seveso second generation: a prospective cohort study. Environ. Health 17, 22. 29482571 10.1186/s12940-018-0365-2 PMC5827999

[R72] Yu G, Wang L-G, Han Y, He Q-Y, 2012. clusterProfiler: an R package for comparing biological themes among gene clusters. OMICS 16 (5), 284–287. 22455463 10.1089/omi.2011.0118 PMC3339379

