# Supplementary material for: Unraveling the differential impact of PAHs and dioxin-like compounds on AKR1C3 reveals the EGFR extracellular domain as a critical determinant of the AHR response
Source: Environ Int. Author manuscript; Available in PMC 2022 Feb 17. (PMC8852774; doi:10.1016/j.envint.2021.106989)
Supplement: word doc [file NIHMS1771411-supplement-word_doc.docx]

Supplementary Information

**Unraveling the differential impact of PAHs and dioxin-like compounds on AKR1C3 reveals the EGFR extracellular domain as a critical determinant of the AHR response**

Christian Vogeley, Natalie C. Sondermann, Selina Woeste, Afaque A. Momin, Viola Gilardino, Frederick Hartung, Markus Heinen, Sophia K. Maaß, Melina Mescher, Marius Pollet, Katharina M. Rolfes, Christoph F.A. Vogel, Andrea Rossi, Dieter Lang, Stefan T. Arold, Motoki Nakamura, Thomas Haarmann-Stemmann

Correspondence to: Thomas.haarmann-stemmann@iuf-duesseldorf.de

This PDF file includes:

Supplementary Figures S1 – S10

Supplementary Table 1 – 2


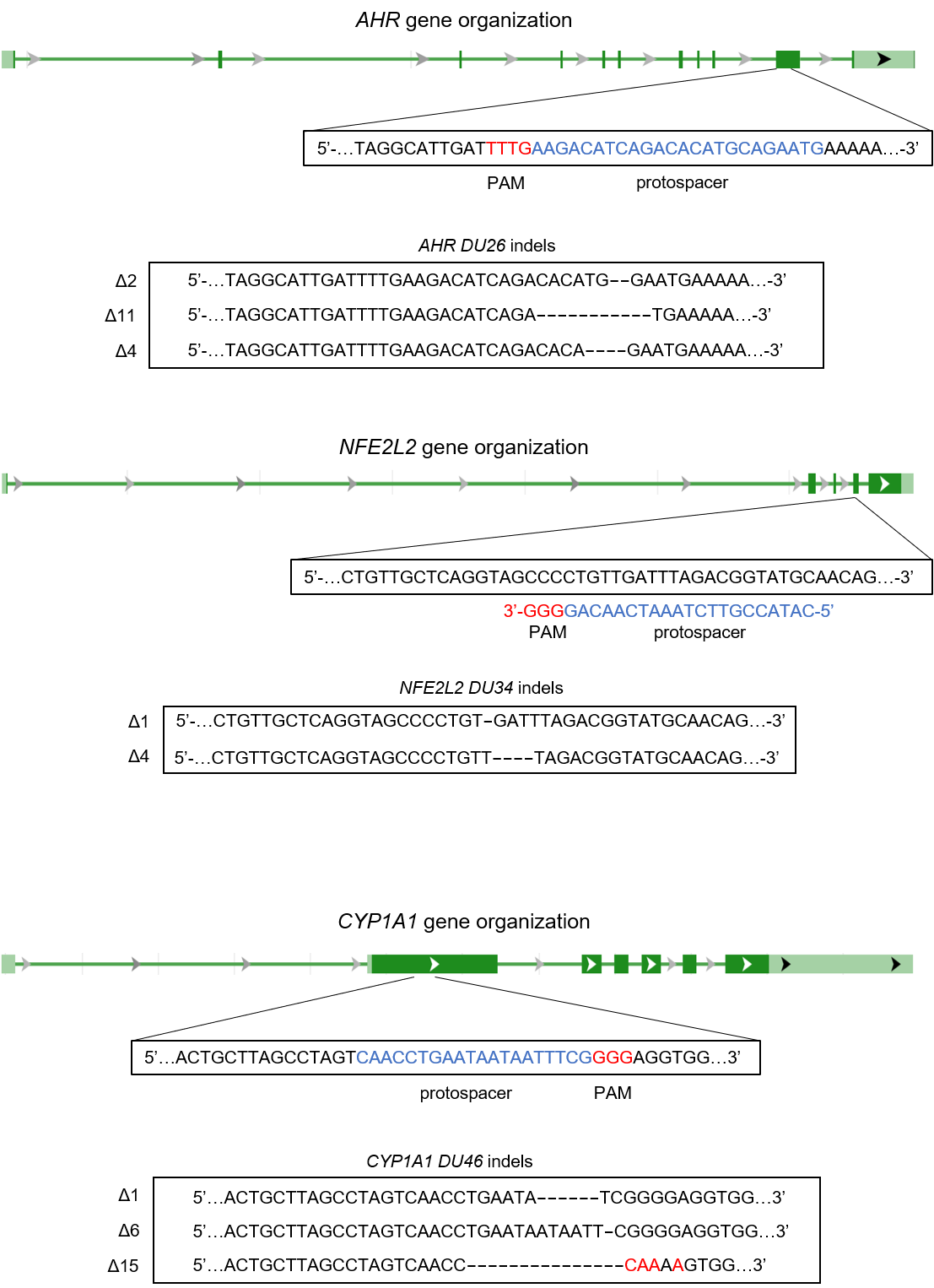


**Figure S1: Generation of AHR-, NRF2-, and CYP1A1-KO HaCaT keratinocytes**

Shown is the organisation of the respective genes, as well as the sequence of the used gRNA with the protospacer adjacent motif depicted in red and the protospacer in blue. The clones were genotyped using high-resolution melt analysis, SANGER sequencing or deep sequencing. The induced indels are shown in the box below.


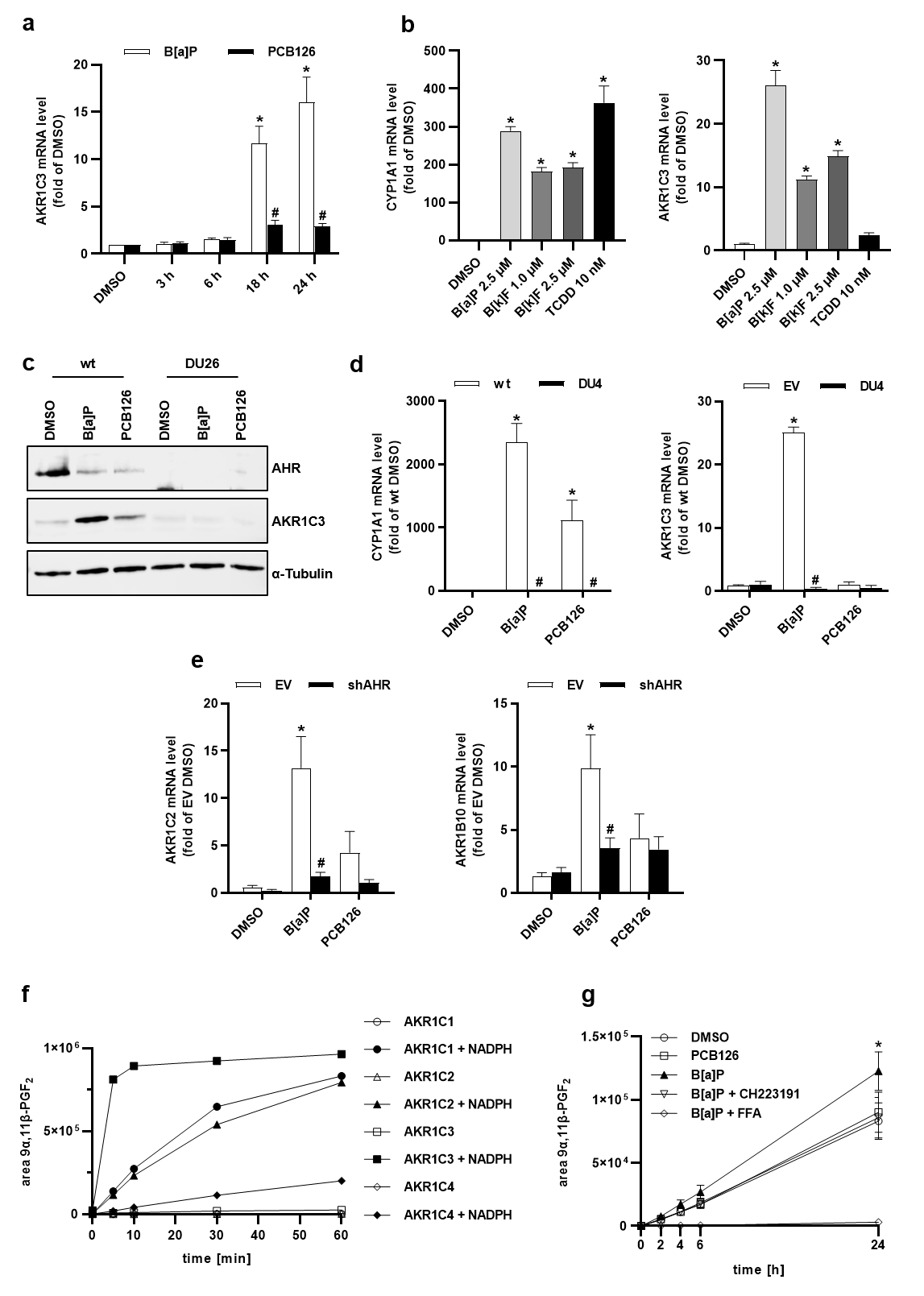


**Figure S2: PAHs but not DLCs induce AKR1C3 in an AHR-dependent manner**

**a** qRT-PCR analyses of *AKR1C3* in HaCaT keratinocytes stimulated with 2.5 µM B[a]P or 1 µM PCB126 for the indicated time. n = 6. *, p ≤ 0.05 compared to the corresponding DMSO control, #, p ≤ 0.05, compared to B[a]P of the time point. **b** qRT-PCR analyses of *CYP1A1* and *AKR1C3* transcript level in HaCaT keratinocytes treated as indicated for 24 h. n = 3. *, p ≤ 0.05 compared to DMSO. **c** Western blot analyses of AHR and AKR1C3 levels in HaCaT-AHR-KO (DU26) and wild-type (wt) controls. Cells were stimulated either with 2.5 µM B[a]P, 1 µM PCB126 or 0.1 % DMSO. α-Tubulin was used as loading control. n = 3, representative picture. **d** qRT-PCR analyses of *CYP1A1* and *AKR1C3* transcript level in MCF-7-AHR-KO (DU4) and wildtype (wt) MCF-7 cells stimulated with 2.5 µM B[a]P, 1 µM PCB126 or 0.1 % DMSO for 24 h. n = 3. *, p ≤ 0.05 compared to EV DMSO, #, p ≤ 0.05 compared to EV B[a]P. **e** qRT-PCR analyses of *AKR1C2* and *AKR1B10* of HaCaT-shAHR and HaCaT-EV keratinocytes exposed to 0.1 % DMSO, 2.5 µM B[a]P or 1 µM PCB126 for 24 h. n = 4. *, p ≤ 0.05 compared to EV DMSO, #, p ≤ 0.05 compared to EV B[a]P. **f** Heterologously expressed AKR1C isoforms were incubated with to 2 µM PGD_2_ in the presence or absence of NADPH. LC-MS was used to analyze metabolically formed 9α,11β-PGF_2_ in the cell culture supernatants at the indicated time points. **g** LC-MS analyses of NHEK-derived culture supernatants. Cells were stimulated with 0.1 % DMSO, 1 µM PCB126, 2.5 µM B[a]P, 2.5 µM B[a]P plus 10 µM CH223191 for 24 h. In addition, keratinocytes pretreated for 23 h with 2.5 µM B[a]P were co-exposed for 1 h to 50 µM flufenamic acid (FFA) and B[a]P. Subsequently, cells were treated with 1 µM PGD_2_ in conditioned medium and the supernatant was collected at indicated time points. n = 4. *, p ≤ 0.05 compared to DMSO.


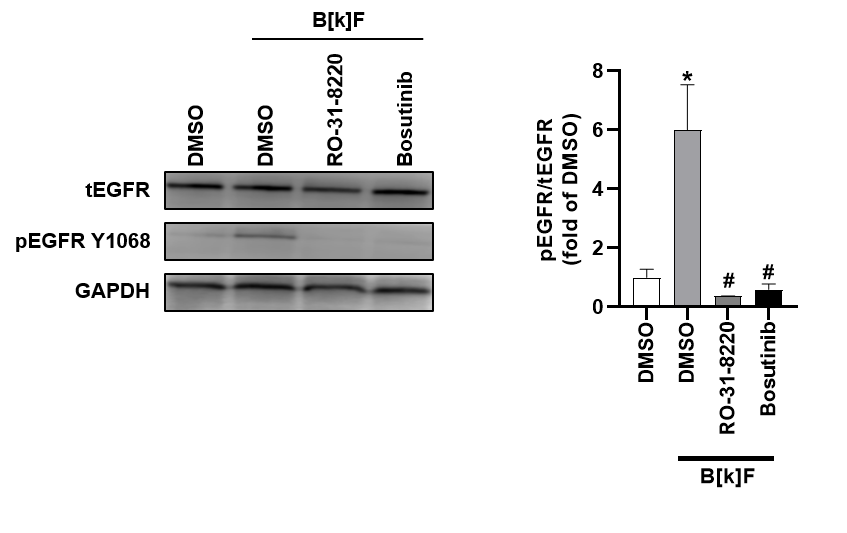


**Figure S3: B[k]F induces phosphorylation of the EGFR**

Western blot analysis of HaCaT cells treated with B[k]F (2.5 µM) for 2 h or control treated with DMSO (0.1 %). In parallel, cells were co-treated with RO-31-8220 (1 µM) or bosutinib (1 µM). Representative western blot of total EGFR and phosphorylated EGFR at residue Y1068. GAPDH was used as endogenous loading control. Densitometric quantification of the western blot analysis is shown in the right panel. n = 3. *, p ≤ 0.05 compared to DMSO, #, p ≤ 0.05 compared to DMSO/B[k]F.


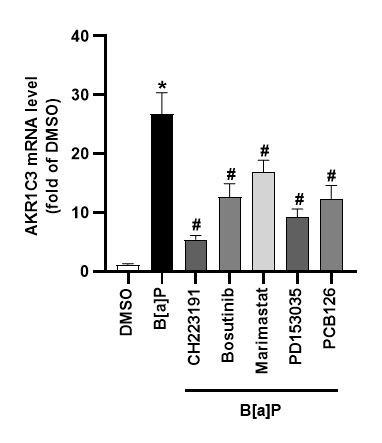


**Figure S4: Induction of *AKR1C3* *via* a non-canonical AHR signaling pathway is conserved in breast cancer cells**

qRT-PCR analyses of *AKR1C3* in MCF-7 cells treated with B[a]P (2.5µM) in the absence or presence of CH223191 (10 µM), Bosutinib (1 µM), Marimastat (1 µM), PD153035 (1 µM), PCB126 (1 µM) or DMSO (0.1 %) for 24 h. n = 8. *, p ≤ 0.05 compared to DMSO, #, p ≤ 0.05 compared to B[a]P.


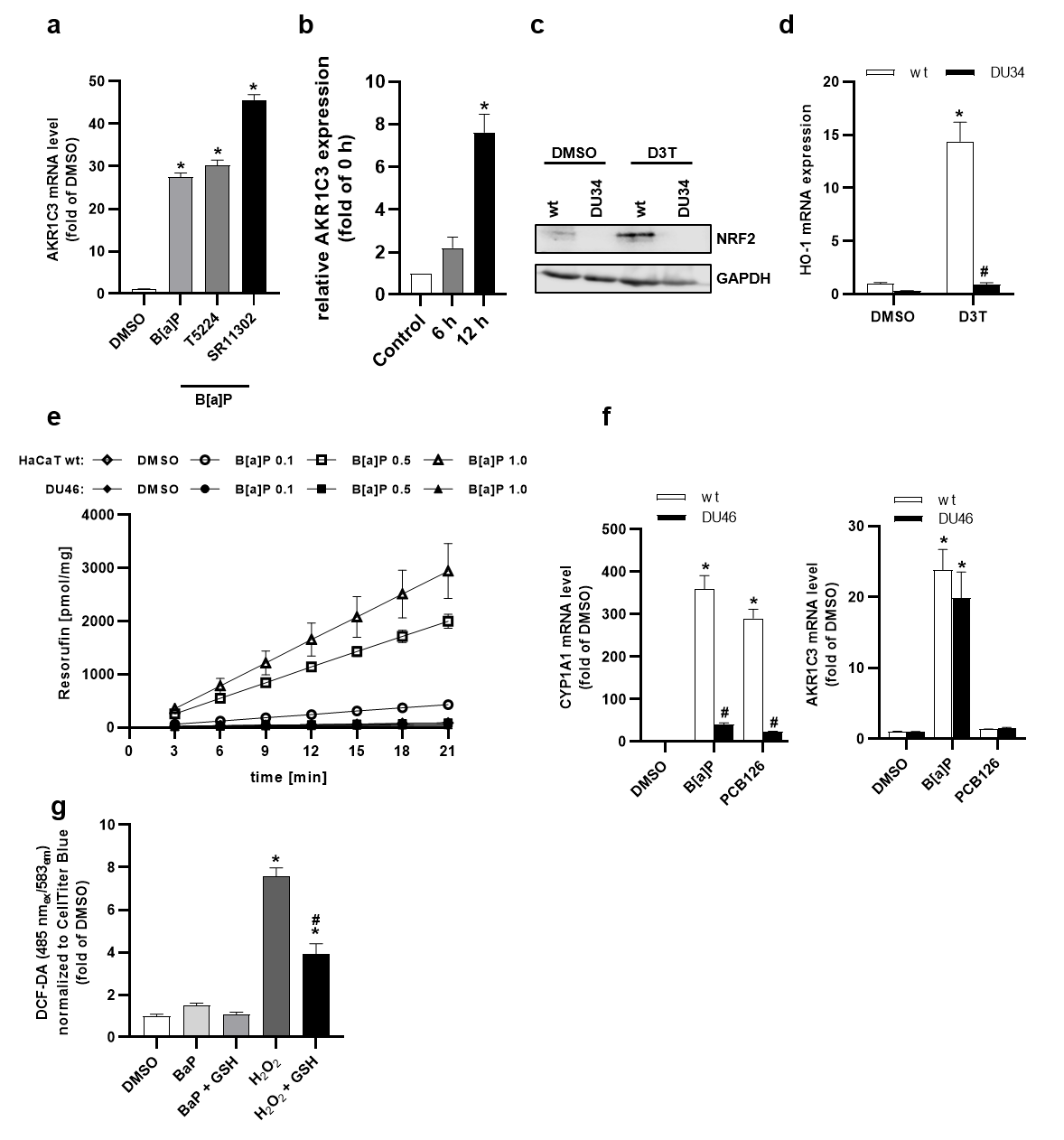


**Figure S5: NRF2 regulates AKR1C3 expression in a ROS-independent manner**

**a** Effect of AP-1 inhibition on AKR1C3 expression was analyzed via qRT-PCR. HaCaT keratinocytes were treated with DMSO (0.1 %), B[a]P (2.5 µM) alone or in combination with the AP-1 inhibitors T5224 or SR11302 (both 10 µM) for 24 h. n = 3. *, p ≤ 0.05 compared to DMSO**. b** qRT-PCR analysis of AKR1C3 in HaCaT keratinocytes. The cells were treated with 2.5 µM B[a]P or 0.1 % DMSO for 6 h and 12 h. n = 3. *, p ≤ 0.05 compared to DMSO **c** Western blot analyses of HaCaT and HaCaT-NRF2-KO (DU34) keratinocytes. Cells were treated for 24 h with DMSO (0.1 %) or D3T (70 µM). n = 1. **d** qRT-PCR analyses of *HO-1* in HaCaT and HaCaT-NRF2-KO (DU34) keratinocytes treated as described in **c.** n = 3. *, p ≤ 0.05 compared to DMSO wt, #, p ≤ 0.05 compared to the respective control sample. **e** HaCaT and HaCaT-CYP1A1-KO (DU46) cells were treated for 24 h with 0.1 µM, 0.5 µM and 1 µM B[a]P. Conversion of 7-ethoxyresorufin to resorufin was measured over 21 min and is shown in pmol/mg protein. **f** qRT-PCR analyses of CYP1A1 and AKR1C3 in HaCaT and HaCaT-CYP1A1-KO (DU46) keratinocytes. Cells were treated with B[a]P (2.5 µM), PCB126 (1 µM) or DMSO (0.1 %) DMSO for 24 h. n = 3. *, p ≤ 0.05 compared to DMSO HaCaT control, #, p ≤ 0.05 compared to the respective proficient HaCaT control. **g** ROS formation was analyzed by DCF-DA assay. HaCaT cells were treated as indicated for 6 h. As a positive control cells were treated with H_2_O_2_ (10 µM) 30 min prior staining. n = 3. *, p ≤ 0.05 compared to DMSO.


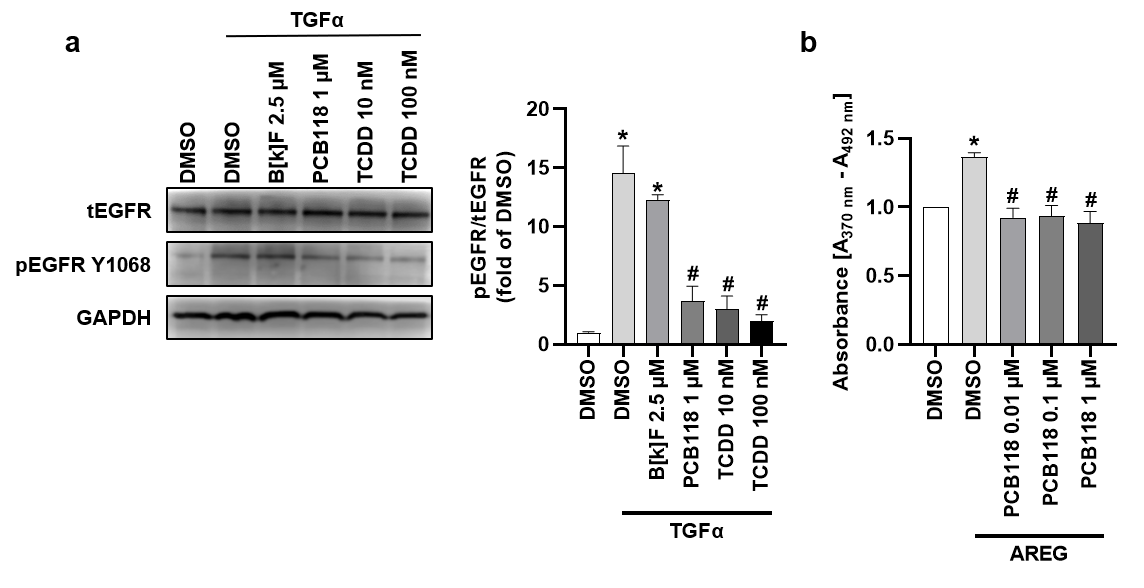


**Fig. S6: Dioxin-like compounds interfere with EGFR ligand-induced EGFR activation**

**a** Effect of B[k]F, PCB118 and TCDD on EGFR activation upon TGFα stimulation was analyzed via western blot analysis. HaCaT keratinocytes were starved for 3 h and next stimulated with TGFα (20 ng/ml) for 2.5 min on ice. Afterwards the cells were treated as indicated and the cells were incubated at 37 °C an 5 % CO2 for 30 min. Levels of total and phosphorylated EGFR (Y1068) were determined; GAPDH was used as endogenous loading control. n = 3. representative pictures are shown in the left panel. Densitometric quantification is depicted in the right panel. n = 3. *, p ≤ 0.05 compared to DMSO. #, p ≤ 0.05 compared to DMSO/AREG. **b** Colorimetric BrdU incorporation assay to assess the influence of PCB118 on AREG-induced DNA synthesis. HaCaT AHR-KO keratinocytes were treated as indicated for 4 h. Absorption was measured at a wavelength of 370 nm (reference wavelength 492 nm). n = 3. *, p ≤ 0.05 compared to DMSO. #, p ≤ 0.05 compared to AREG/DMSO.

**
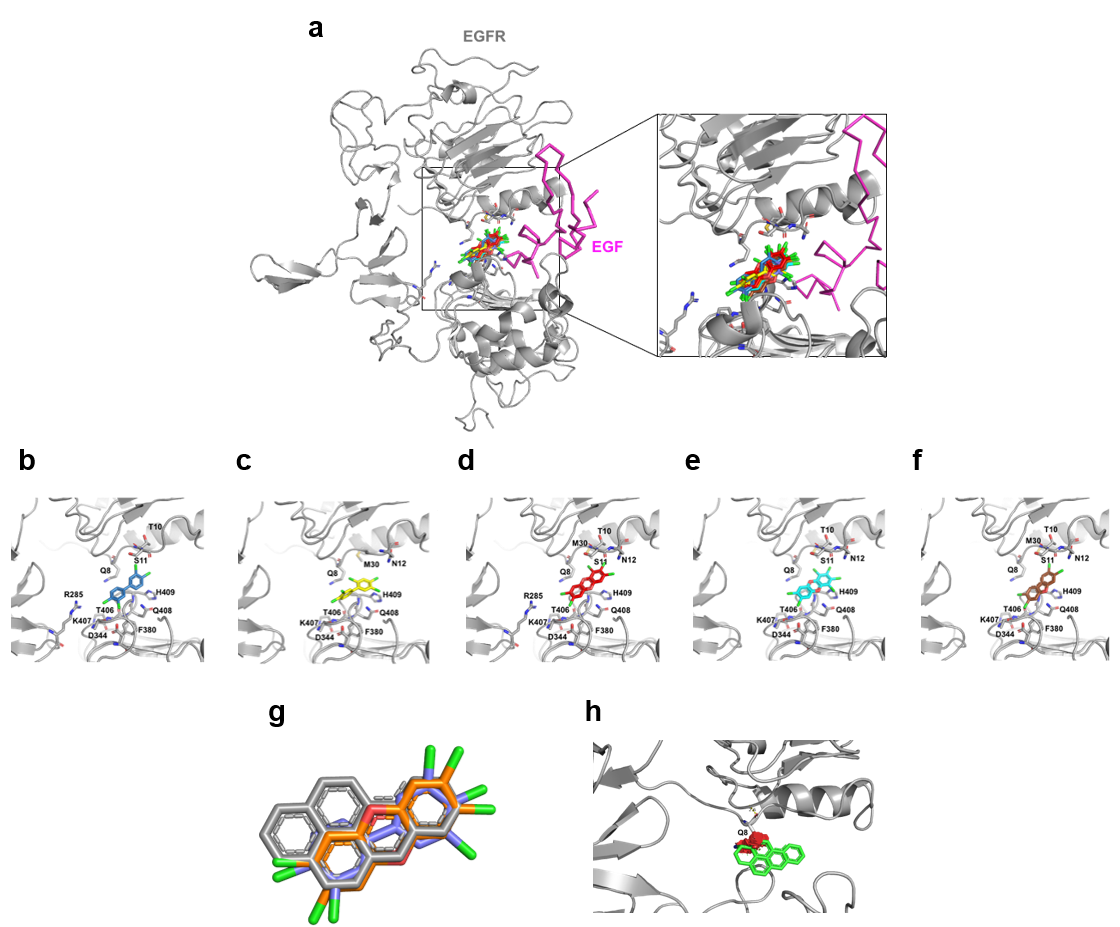
**

**Fig. S7: Dioxin-like compounds bind to the extracellular domain of the EGFR.**

**a** *In silico* docking analyses predicting the binding of PCB126 (slate blue), TCDD (orange), PCB77 (blue), PCB118 (yellow), PCDD (red), HCDD (cyan), and TCDF (brown) to the extracellular domain (ECD) of EGFR (grey). EGF (magenta; taken from PDB ID: 1ivo) is superimposed. Interacting residues for EGFR to the ligands are shown in stick representation. Interacting amino acid residues of EGFR ECD for **b** PCB77, **c** PCB118, **d** PCDD, **e** HCDD and **f** TCDF shows as stick models. **g** Superimposed compound structures of PCB126 (slate blue), TCDD (orange) and B[a]P (gray) shown as stick models. **h** B[a]P (green) would clash with EGFR Q8 in the same position as PCB126.


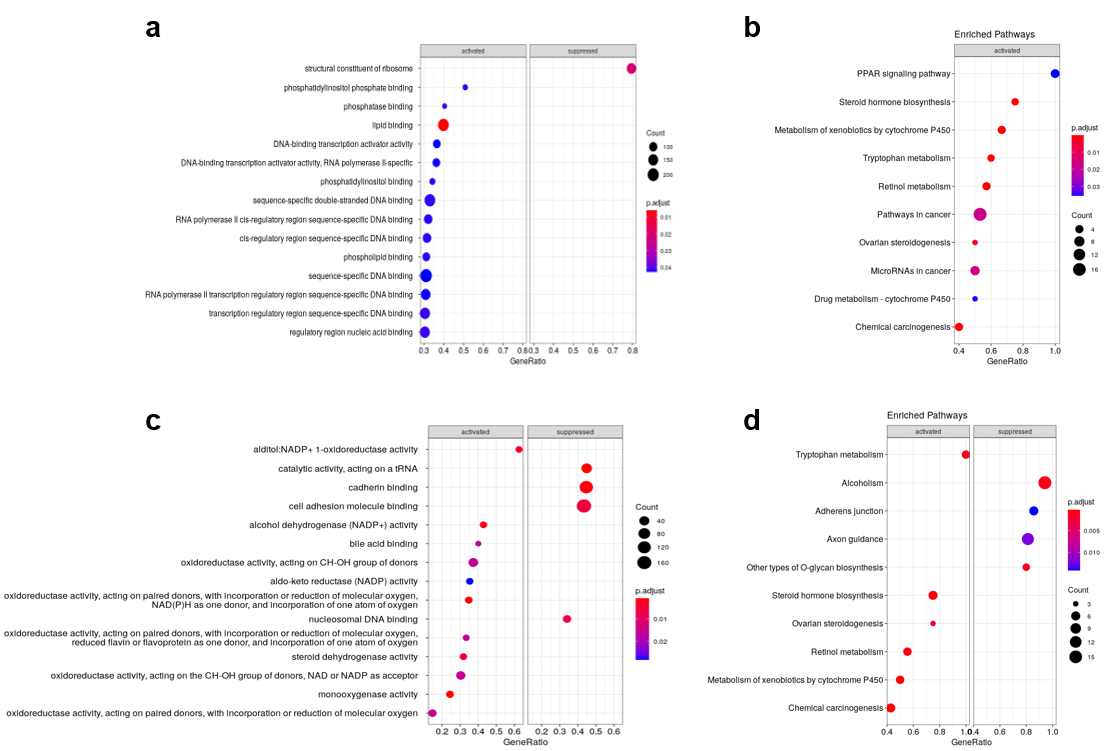


**Figure S8: Gene set enrichment analyses of PCB126 and B[a]P treated HaCaT keratinocytes**

Samples and data shown in Fig. 7 were used for these analyses. PCB126 treated HaCaT keratinocytes were compared to control treated cells (DMSO 0.1 %) and **a** gene set enrichment analysis of molecular functions and **b** KEGG pathway analysis were performed. **c** Gene set enrichment analysis of molecular functions and **d** KEGG pathway analysis of B[a]P treated samples compared to control treated cells (DMSO 0.1 %).


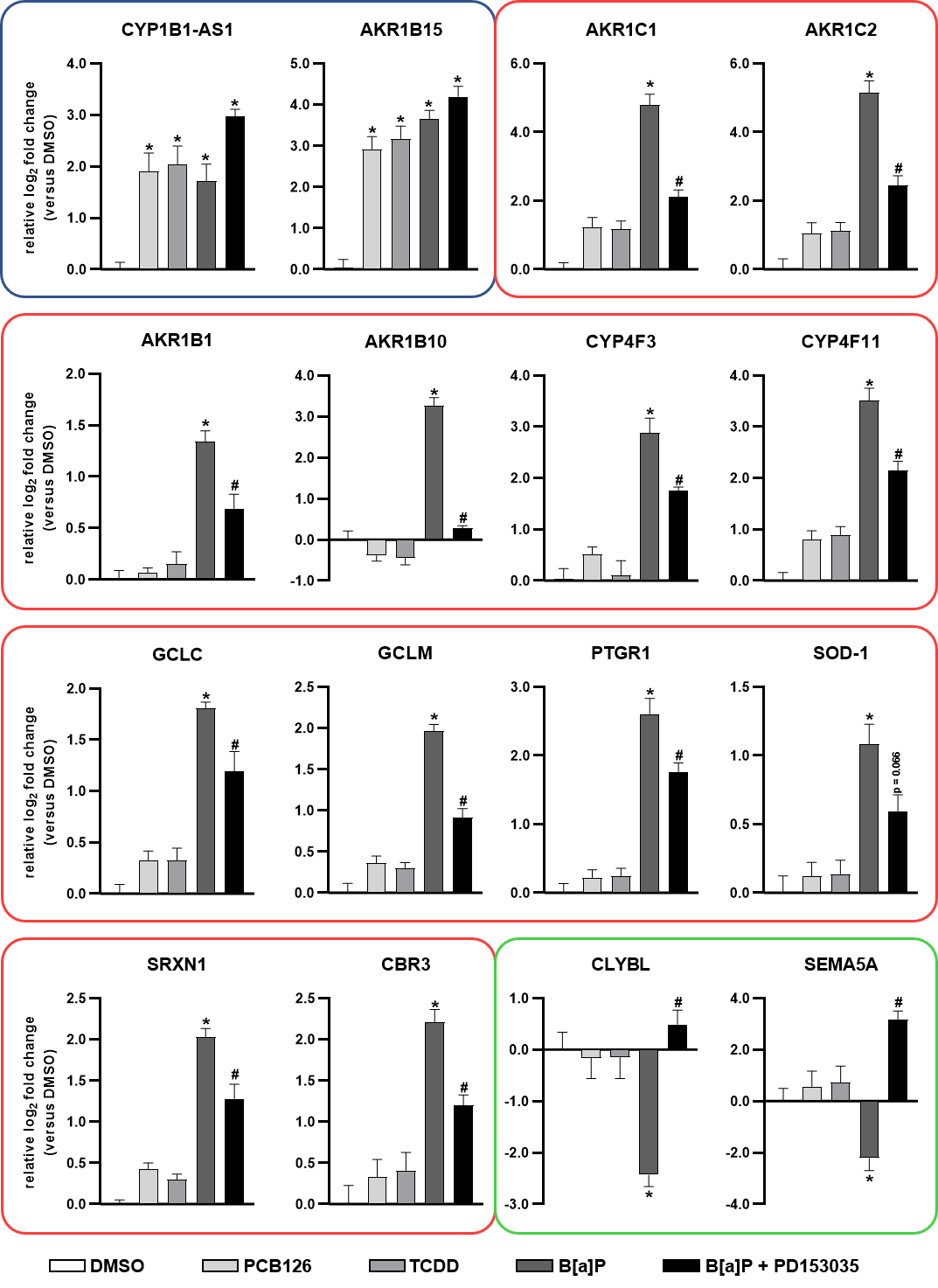


**Figure S9: Ligand-specific gene expression pattern in HaCaT keratinocytes**

qRT-PCR analyses of selected genes in HaCaT cells. Keratinocytes were treated for 24 h with either DMSO (0.1 %), PCB126 (1 µM), TCDD (10 nM), B[a]P (2.5 µM) or B[a]P (2.5 µM) + PD153035 (1 µM). Results were normalized to DMSO-treated samples and log_2_ transformed. n = 4. *, p ≤ 0.05 compared to DMSO, #, p ≤ 0.05 compared to B[a]P.

Human LEEKKVC**Q**G**TSN**KLTQLGTFEDHFLSLQR**M**FNNCEVVLGNLEITYVQRNYDLSFLKTIQE 60

Rhesus_monkey LEEKKVC**Q**G**TSN**KLTQLGTFEDHFLSLQR**M**FNNCEVVLGNLEITYVQRNYDLSFLKTIQE 60

Marmoset LEEKKVC**Q**G**TSN**KLTQLGTFEDHFLSLQR**M**FNNCEVVLGNLEITYVQRNYDLSFLKTIQE 60

Mouse LEEKKVC**Q**G**TSN**RLTQLGTFEDHFLSLQR**M**YNNCEVVLGNLEITYVQRNYDLSFLKTIQE 60

Rat LEEKKVC**Q**G**TSN**RLTQLGTFEDHFLSLQR**M**FNNCEVVLGNLEITYVQRNYDLSFLKTIQE 60

Rabbit LEEKRVC**Q**G**TSN**KLTQLGPFEDHFLSLQR**M**FNSCEVVLGNLEITYVQRNHDLSFLKTIQE 60

Pig LEEKKVC**Q**G**TSN**KLTQLGTFEDHFLSLQR**M**FNNCEVVLGNLEITYMQNSYNLSFLKTIQE 60

Sheep LEEKKVC**Q**G**TSN**KLTQLGTFEDHFLSLQR**M**FNNCEVVLGNLEITYMQSGYNLSFLKTIQE 60

Cat LEEKKVC**Q**G**TSN**RLTQLGTFEDHFLSLQR**M**FNNCEVVLGNLEITYMQRNYDLSFLKTIQE 60

Dog LEEKRVC**Q**G**TSN**RLTQLGTFEDHFLSLQR**M**FNNCEVVLGNLEITYMQRNYDLSFLKTIQE 60

****:*******:***** ***********:*.************:* .::*********

Human VAGYVLIALNTVERIPLENLQIIRGNMYYENSYALAVLSNYDANKTGLKELPMRNLQEIL 120

Rhesus_monkey VAGYVLIALNTVERIPLENLQIIRGNMYYENSYALAVLSNYDANKTGLKELPMRNLQEIL 120

Marmoset VAGYVLIALNTVERIPLENLQIIRGNMFYENSYALAVLSNYDANKTGLKELPMRNLQEIL 120

Mouse VAGYVLIALNTVERIPLENLQIIRGNALYENTYALAILSNYGTNRTGLRELPMRNLQEIL 120

Rat VAGYVLIALNTVERIPLENLQIIRGNALYENTYALAVLSNYGTNKTGLRELPMRNLQEIL 120

Rabbit VAGYVLIALNTVESIPLESLQIIRGNVLYENTYALAVLSNYGANKTGLRELPMRNLQEIL 120

Pig VAGYVLIALNTVEKIPLENLQIIRGNVLYENTHALAVLSNYGANKTGLRELPMRNLQEIL 120

Sheep VAGYVLIALNTVEKIPLENLQIIRGNVLYENTHALAVLSNYGANKTGLRELPLRNLQEIL 120

Cat VAGYVLIALNTVEKIPLENLQIIRGNVLYENTHALSVLSNYGTNKTGLRELPMRNLHEIL 120

Dog VAGYVLIALNTVEKIPLENLQIIRGNVLYENTHALSVLSNYGSNKTGLQELPLRNLHEIL 120

************* ****.******* ***::**::****.:*:***:***:***:***

Human HGAVRFSNNPALCNVESIQWRDIVSSDFLSNMSMDFQNHLGSCQKCDPSCPNGSCWGAGE 180

Rhesus_monkey HGAVRFSNNPALCNVESIQWRDIVSSEFLSNMSMDFQNHLGSCQKCDPSCPNGSCWGAGE 180

Marmoset HGAVRFSNNPTLCNVDSIQWRDIVSNDFLSNMSMDFQNHVAGCQKCDPSCPNGSCWGAGE 180

Mouse IGAVRFSNNPILCNMDTIQWRDIVQNVFMSNMSMDLQSHPSSCPKCDPSCPNGSCWGGGE 180

Rat IGAVRFSNNPILCNMETIQWRDIVQDVFLSNMSMDVQRHLTGCPKCDPSCPNGSCWGRGE 180

Rabbit HGAVRFSNNPVLCNVETIQWRDIVHGDFLSNMSLDMQNPLGSCQKCDPSCPNGSCWGAGG 180

Pig QGAVRFSNNPALCHAESIQWRDIVNSDFLSNMSMDFQSQLGSCPKCDPGCLNGSCWGAGK 180

Sheep QGAVRFSNNPVLCNMETIQWRDIINTDFLSNVTGDFQNQQGNCSKCDPGCLNRSCWGAGE 180

Cat QGAVRFSNNPVLCNVETIQWRDIVDNDFISNMSMDFQNHVGNCQKCDPGCPNGSCWGPGK 180

Dog QGAVRFSNNPVLCNVETIQWRDIVDNDFISNMSMDIQNQAGRCQKCDPSCPNGSCWGPGK 180

********* **: ::******: *:**:: *.* * ****.* * **** *

Human ENCQKLTKIICAQQCSGRCRGKSPSDCCHNQCAAGCTGPRESDCLVCRKFRDEATCKDTC 240

Rhesus_monkey ENCQKLTKIICAQQCSGRCRGKSPSDCCHNQCAAGCTGPRESDCLVCRKFRDEATCKDTC 240

Marmoset ENCQKLTKIICAQQCSGRCRGKSPSDCCHNQCAAGCTGPRESDCLVCRKFRDEATCKDTC 240

Mouse ENCQKLTKIICAQQCSHRCRGRSPSDCCHNQCAAGCTGPRESDCLVCQKFQDEATCKDTC 240

Rat ENCQKLTKIICAQQCSRRCRGRSPSDCCHNQCAAGCTGPRESDCLVCHRFRDEATCKDTC 240

Rabbit ENCQRLTKIICAQQCSGRCRGKSPSDCCHNQCAAGCTGPRESDCLVCRKFRDEATCKDTC 240

Pig ENCQKLTKVICAQQCSGRCRGRSPSDCCHNQCAAGCTGPRESDCLVCRRFRDEATCKDTC 240

Sheep ENCQKLTKIICAQQCSGRCRGRSPSDCCHNQCAAGCTGPRESDCLVCRRFRDEATCKDTC 240

Cat ENCQKLTKIICAQQCSGRCRGRSPSDCCHNQCAAGCTGPRESDCLVCRKFRDEATCKDTC 240

Dog ENCQKLTKIICAQQCSGRCRGRSPSDCCHNQCAAGCTGPRESDCLVCRKFRDEATCKDTC 240

****:***:******* ****:*************************::*:*********

Human PPLMLYNPTTYQMDVNPEGKYSFGATCVKKCPRNYVVTDHGSCV**R**ACGADSYEMEEDGVR 300

Rhesus_monkey PPLMLYNPTTYQMDVNPEGKYSFGATCVKKCPRNYVVTDHGSCV**R**ACGADSYEMEEDGVR 300

Marmoset PPLMLYNPTTYQMDVNPEGKYSFGATCVKKCPRNYVVTDHGSCV**R**ACGADSYEVEEDGVR 300

Mouse PPLMLYNPTTYQMDVNPEGKYSFGATCVKKCPRNYVVTDHGSCV**R**ACGPDYYEVEEDGIR 300

Rat PPLMLYNPTTYQMDVNPEGKYSFGATCVKKCPRNYVVTDHGSCV**R**ACGPDYYEVEEDGVS 300

Rabbit PPLMLYNPTTYQMDVNPEGKYSFGATCVKKCPRNYVVTDHGSCV**R**ACGPDSYEVEEDGVR 300

Pig PPLMLYNPTTYQMDVNPLGKYSFGATCVKKCPRNYVVTDHGSCV**R**ACSSDSYEVEEDGVR 300

Sheep PPLMLYDPTTYEMKVNPLGKYSFGATCVKKCPRNYVVTDHGSCV**R**ACSSDSQEVEEDGVR 300

Cat PPLMLYNPTTYQMDVNPEGKYSFGATCVKKCPRNYVVTDHGSCV**R**ACSSDSYEVEEDGVR 300

Dog PPLMLYNPTTYQMDVNPEGKYSFGATCVKKCPRNYVVTDHGSCV**R**ACSSDSYEVEEDGVR 300

******:****:*.*** *****************************. * *:****:

Human KCKKCEGPCRKVCNGIGIGEFKDSLSINATNIKHFKNCTSISG**D**LHILPVAFRGDSFTHT 360

Rhesus_monkey KCKKCEGPCRKVCNGIGIGEFKDTLSINATNIKHFKNCTSISG**D**LHILPVAFRGDSFTHT 360

Marmoset KCKKCEGPCRKVCNGIGIGKFKDTLSINATNIKHFKNCTSISG**D**LHILPVAFRGDSFTHT 360

Mouse KCKKCDGPCRKVCNGIGIGEFKDTLSINATNIKHFKYCTAISG**D**LHILPVAFKGDSFTRT 360

Rat KCKKCDGPCRKVCNGIGIGEFKDTLSINATNIKHFKYCTAISG**D**LHILPVAFKGDSFTRT 360

Rabbit KCKKCEGPCRKVCNGIGIGEFKDTLSINATNIKHFKNCTSISG**D**LHILPVAFRGDSFTRT 360

Pig KCKKCDGPCGKVCNGIGIGEFKDTLSINATNIKHFRNCTSISG**D**LHILPVAFRGDSFTRT 360

Sheep KCKKCDGPCAKVCNGIGIGEFKDTLSINATNIKHFRNCTSISG**D**LHILPVAFRGDSFTRT 360

Cat KCKKCEGPCRKVCNGIGIGEFKDTLSINATNIKHFKNCTSISG**D**LHILPVAFRGDSFTHT 360

Dog KCKKCEGPCRKVCNGIGIGEFKDTLSINATNIKHFKNCTSISG**D**LHILPVAFRGDSFTHT 360

*****:*** *********:***:***********: **:************:*****:*

Human PPLDPQELDILKTVKEITG**F**LLIQAWPENRTDLHAFENLEIIRGR**TKQH**GQFSLAVVSLN 420

Rhesus_monkey PPLDPQELDILKTVKEITG**F**LLIQAWPENRTDLHAFENLEIIRGR**TKQH**GQFSLAVVSLN 420

Marmoset PPLDPKELDILKTVKEITG**F**LLIQAWPENRTDLHAFENLEIIRGR**TKQH**GQFSLAVVSLN 420

Mouse PPLDPRELEILKTVKEITG**F**LLIQAWPDNWTDLHAFENLEIIRGR**TKQH**GQFSLAVVGLN 420

Rat PPLDPRELEILKTVKEITG**F**LLIQAWPENWTDLHAFENLEIIRGR**TKQH**GQFSLAVVGLN 420

Rabbit PPLDPEELDILKTVREITG**F**LLIQAWPENKTDLHAFENLEIIRGR**TKQH**GQFSLAVVGLD 420

Pig PPLDPKELDILKTVKEITG**F**LLIQAWPENRTGLHAFENLEIIRGR**TKQH**GQFSLAVVGLD 420

Sheep APLDPKELDILKTVKEITG**F**LLIQAWPENRTDLHAFENLEIIRGR**TKQH**GQFSLAVVGLD 420

Cat PPLDPKELDILKTVKEITG**F**LLIQAWPENRTDLHAFENLEIIRGR**TKQH**GQFSLAVVGLD 420

Dog LPLDPKELDILKTVKEITG**F**LLIQAWPENRTDLHAFENLEIIRGR**TKQH**GQFSLAVVGLN 420

****.**:*****:************:* *.*************************.*:

**Supplementary figure S10: Multiple alignment of mammalian EGFR protein sequences.**

A multiple alignment of the N-terminal 420 amino acids of the mammalian EGFR protein (mature) was carried out using the Clustal Omega online tool and the NCBI References Sequences given in the Material and Methods section. Bold letters in blue stand for amino acid residues predicted to be involved in the binding of all tested DLCs. Bold letter in purple stand for amino acid residues predicted to be involved in the binding of some of the test compounds. Bold letters in green stand for predicted amino acid residues that were experimentally proven to be required for binding of PCB126. Bold letters in red stand for predicted amino acids that were experimentally excluded being critically required for DLC binding. “*” = fully conserved residue, “:” = conservation between groups of strongly similar properties, “.” = conservation between groups of weakly similar properties.

**Supplementary table 1**

| **A) qPCR oligonucleotides** | |  |  |
| --- | --- | --- | --- |
|  |  |  |  |
| Gene symbol | Gene | Direction | Sequence (5' > 3') |
| ACTB | β-Actin | F | CCCCAGGCACCAGGGCGTGAT |
|  |  | R | GGTCATCTTCTCGCGGTTGGCCTTGGGGT |
| AKR1B1 | Aldo-keto reductase 1B1 | F | TACCATGAGAAGGGCCTGGTGAAA |
|  |  | R | TCCAGAATGTTGGTGTCACTGGGA |
| AKR1B10 | Aldo-keto reductase 1B10 | F | TTCTTTGAGAGACCCCTTGTGAGG |
|  |  | R | TCCAAGAACGTTGCTTTTCCACCG |
| AKR1B15 | Aldo-keto reductase 1B15 | F | ACTGGCCTAAAGAGTTCCCTTC |
|  |  | R | GCATCAATGGCCACCTTCAC |
| AKR1C1 | Aldo-keto reductase 1C1 | F | CAATTGAAGCTGGCTTCCGC |
|  |  | R | TCACTTCCTCACCTGGCTTT |
| AKR1C2 | Aldo-keto reductase 1C2 | F | TCTAGAGGCCGTCAAATTGGCA |
|  |  | R | TGGTCGATGGGAATTGCTCCAA |
| AKR1C3 | Aldo-keto reductase 1C3 | F | AAGCTGGGTTCCGCCATATAGA |
|  |  | R | CAGTGAGTTTTCCAAGGCTGG |
| ARHGAP26 | Rho GTPase activating protein 26 | F | TAAGAATGCTTCCAGGACCACTC |
|  |  | R | GCTGTAACATCTGCCGATTTTTC |
| CBR3 | Carbonyl reductase 3 | F | AGTTCTCTGGGGATGTGGTG |
|  |  | R | GTTGACCAGTACGTTGAGCC |
| CLYBL | Citrate lyase beta like | F | TCGACTGTGAGGATGGAGTG |
|  |  | R | ATCAGGCTGGAAGGAAGGAC |
| CYP1A1 | Cytochrome P450 1A1 | F | TTCATGCAGAAGATGGTC |
|  |  | R | TCTCCTGACAGTGCTCAATC |
| CYP1B1 | Cytochrome P450 1B1 | F | CGGCTGGATTTGGAGAACGTA |
|  |  | R | TGATCCAATTCTGCCTGCACT |
| CYP1B1-AS1 | CYP1B1-antisense RNA 1 | F | GTGCAGTTGTGAAGTCAGCA |
|  |  | R | ATGAGCATGGAGAAGGGAGG |
| CYP4F3 | Cytochrome P450 4F3 | F | GAGGAGGTTGTGTGGGACAAGG |
|  |  | R | GTGGAAGATGCGGACGATTGCG |
| CYP4F11 | Cytochrome P450 4F11 | F | CGAAACAGAACTGGTTTTGGG |
|  |  | R | GGTCAATGTCTTCATGCCCTC |
| GAPDH | Glyceraldehyde 3-phosphate | F | AGGTGAAGGTCGGAGTCA |
|  | dehydrogenase | R | GGTCATTGATGGCAACAA |
| GCLC | Glutamate-cysteine ligase | F | GGCACAAGGACGTTCTCAAGT |
|  | catalytic subunit | R | CAGACAGGACCAACCGGAC |
| GCLM | Glutamate-cysteine ligase | F | CATTTACAGCCTTACTGGGAGG |
|  | modifier subunit | R | ATGCAGTCAAATCTGGTGGCA |
| HO-1 | Heme oxygenase 1 | F | GCCATGAACTTTGTCCGGTG |
|  |  | R | GGATGTGCTTTTCGTTGGGG |
| PTGR1 | Prostaglandin reductase 1 | F | TGGCCAGACACAATACCACT |
|  |  | R | CAGACCCTACTGCTCCAACA |
| SEMA5A | Semaphorin 5a | F | GTCTATACTTACTGCCAGCG |
|  |  | R | GTTAAATGCCTTGATGGCCTC |
| SOD1 | Superoxide dismutase 1 | F | GGCCGATGTGTCTATTGAAGA |
|  |  | R | GGGCCTCAGACTACATCCAA |
| SRXN1 | Sulfiredoxin 1 | F | CAAGGTGCAGAGCCTCGT |
|  |  | R | CAGCCCCCAAAGGAGTAGAA |

| **B) Primary antibodies** | |  |
| --- | --- | --- |
|  |  |  |
| Antibody | Supplier | Catalog No. |
| β-Actin | Cell Signaling Technology, Frankfurt a.M., Germany | 3700 |
| AHR | Cell Signaling Technology, Frankfurt a.M., Germany | 83200 |
| AKR1C3 | R&D Systems, Minneapolis, MI, USA | MAB7678 |
| ARNT | Santa Cruz Biotechnology, Dallas, TX, USA | sc-55526 |
| CYP1A1 | Santa Cruz Biotechnology, Dallas, TX, USA | sc-20772 |
| EGFR | Cell Signaling Technology, Frankfurt a.M., Germany | 2232 |
| pEGFR Y845 | Cell Signaling Technology, Frankfurt a.M., Germany | 2231 |
| pEGFR Y1068 | Cell Signaling Technology, Frankfurt a.M., Germany | 3777 |
| pEGFR Y1173 | Cell Signaling Technology, Frankfurt a.M., Germany | 4407 |
| ERK1/2 | Cell Signaling Technology, Frankfurt a.M., Germany | 9102 |
| pERK1/2 T202/Y204 | Cell Signaling Technology, Frankfurt a.M., Germany | 9101 |
| GAPDH | Cell Signaling Technology, Frankfurt a.M., Germany | 2118 |
| NRF2 | GeneTex, Irvine, CA, USA | GTX103322 |
| Src | Upstate, Lake Placid, NY, USA | 05-184 |
| pSrc Y416 | Cell Signaling Technology, Frankfurt a.M., Germany | 6943 |
| α-Tubulin | ExBio, Vestec, Czech Republic | 11-250-C025 |
| Vinculin | Cell Signaling Technology, Frankfurt a.M., Germany | 13901 |

| **C) Mutagenesis oligonucleotides** | | |
| --- | --- | --- |
|  |  |  |
| AA exchange | Direction | Sequence (5' > 3') |
| Q8A | F | GAAAAGAAAGTTTGCGCAGGCACGAGTAACAAG |
|  | R | CTTTTCTTTCAAACGCGTCCGTGCTCATTGTTC |
| S11A | F | GTTTGCCAAGGCACGGCTAACAAGCTCACGCAG |
|  | R | CAAACGGTTCCGTGCCGATTGTTCGAGTGCGTC |
| Q408A | F | CGCGGCAGGACCAAGGCACATGGTCAGTTTTCT |
|  | R | GCGCCTGGTTCCGTGTACCAGTAAAAGA |

**Supplementary table 2:** Docking score, calculated affinity and residues involved for all AHR ligands tested with the ECD of EGFR.

| **Ligand name** | **Docking Score**  **(kcal/mol)** | **Calculated**  **affinity (nM)** | **Residues involved** |
| --- | --- | --- | --- |
| TCDD | -11.6 | 55.7 | Q8, T10, S11, N12, M30, D344, F380, T406, K407, Q408, H409 |
| PCB126 | -11.1 | 59.3 | Q8, T10, S11, N12, M30, R285, D344, F380, T406, K407, Q408, H409 |
| PCB77 | -10.1 | 67.9 | Q8, T10, S11, R285, D344, F380, T406, K407, Q408, H409 |
| PCB118 | -9.3 | 78.6 | Q8, N12, M30, D344, F380, T406, K407, Q408, H409 |
| PCDD | -11.5 | 56.4 | Q8, T10, S11, N12, M30, R285, D344, F380, T406, K407, Q408, H409 |
| HCDD | -11.9 | 51.2 | Q8, T10, S11, N12, M30, D344, F380, T406, K407, Q408, H409 |
| TCDF | -11.5 | 55.9 | Q8, T10, S11, N12, M30, D344, F380, T406, K407, Q408, H409 |
| B[a]P | - | - | No interaction at binding site |
